# Advances in Viral Aquatic Animal Disease Knowledge: The Molecular Methods’ Contribution

**DOI:** 10.3390/biology12030466

**Published:** 2023-03-19

**Authors:** Enrico Volpe, Francesca Errani, Luciana Mandrioli, Sara Ciulli

**Affiliations:** Department of Veterinary Medical Sciences, Alma Mater Studiorum, University of Bologna, 47042 Cesenatico, FC, Italy

**Keywords:** *Herpesvirales*, *Iridoviridae*, sturgeon NCLDVs, molecular methods, next generation sequencing, *Nodaviridae*, PCR-based methods, isothermal amplification techniques, sequencing, in situ hybridisation

## Abstract

**Simple Summary:**

Viruses are pervasive components of aquatic ecosystems, and most of them are harmless to humans and animals; however, several aquatic viruses can infect animals, leading to diseases, especially when fish are confined, such as in aquaculture facilities. Traditional methods used to detect and study viruses have been widely applied to aquatic animals’ viruses, leading to the successful isolation, identification and understanding of several of them. However, they have limits, which can be overcome by molecular methods, such as polymerase chain reaction (PCR)-based assays, sequencing and in situ hybridisation. A standard PCR, followed by the sequencing of purified amplicons, is an effective method for both identifying well-known viruses and discovering new ones. In situ hybridisation, in which a labelled probe binds to a nucleic acid sequence in tissue, is able to correlate the presence of viruses to lesions. Novel molecular isothermal methods, such as loop-mediated isothermal amplification (LAMP), were also developed and applied to viral aquatic animal diseases, bringing molecular diagnosis into the field. This review considers the scientific literature dealing with the molecular methods employed hitherto to study the most relevant finfish and shellfish viral pathogens, stressing their advantages and disadvantages.

**Abstract:**

Aquaculture is the fastest-growing food-producing sector, with a global production of 122.6 million tonnes in 2020. Nonetheless, aquatic animal production can be hampered by the occurrence of viral diseases. Furthermore, intensive farming conditions and an increasing number of reared fish species have boosted the number of aquatic animals’ pathogens that researchers have to deal with, requiring the quick development of new detection and study methods for novel unknown pathogens. In this respect, the molecular tools have significantly contributed to investigating thoroughly the structural constituents of fish viruses and providing efficient detection methods. For instance, next-generation sequencing has been crucial in reassignment to the correct taxonomic family, the sturgeon nucleo-cytoplasmic large DNA viruses, a group of viruses historically known, but mistakenly considered as iridoviruses. Further methods such as in situ hybridisation allowed objectifying the role played by the pathogen in the determinism of disease, as the cyprinid herpesvirus 2, ostreid herpesvirus 1 and betanodaviruses. Often, a combination of molecular techniques is crucial to understanding the viral role, especially when the virus is detected in a new aquatic animal species. With this paper, the authors would critically revise the scientific literature, dealing with the molecular techniques employed hitherto to study the most relevant finfish and shellfish viral pathogens.

## 1. Introduction

The advent of molecular methods has benefited all sectors of biology, expanding and deepening knowledge on the structures and operating mechanisms of all organisms. Molecular methods are powerful tools in the study of infectious diseases. They can provide valuable epidemiological insights, demonstrating, for example, relatedness between strains; furthermore, they have contributed to our understanding of pathogenesis and they are invaluable tools in the diagnosis of infectious diseases, expediting and improving pathogen detection and identifying the agents of diseases whose causes are unknown [1].

Polymerase chain reaction (PCR) is the most well-developed molecular technique and, since its invention by Kary Mullis in 1985, it has been used to create many microorganism detection methods and has been applied to all scientific sectors [2]. In PCR, a small amount of DNA can be copied in large quantities over a short period of time, achieving impressive sensitivity. PCR-based methods may overcome the limitations of traditional testing procedures. As a matter of fact, the virology sector profited handsomely from the molecular approach, especially for those viruses with no or few in vitro replication activities. Furthermore, the extensive use of molecular methods has now produced techniques that are also cost-effective when compared to traditional methods. All these features make the molecular approach ideal for the detection of viral aquatic animal diseases. The aquaculture sector is fast expanding and deals with multiple fish species, ranging across the animal kingdom and including freshwater and marine finfish, crustaceans and molluscs. In oceanic marine habitats, viruses can be found in concentrations of up to 10^8^ viruses mL^−1^ [3] and some of them are well-known causes of fish diseases. However, the intensification of fish production and the movement of aquatic animals led to an increase in infectious diseases, requiring a continuous expansion of knowledge asset on fish viral diseases and a constant adjustment of diagnostic methods. In this respect, molecular methods provide the ideal tool to gain crucial knowledge about viral aquatic animal diseases.

The PCR approach has been expanded to detect multiple targets at the same time using multiplexing assays, which has found widespread application in the study and detection of viral aquatic animal diseases. Differential diagnosis, including clinical methods and gross pathology, is a milestone in the diagnosis process for infectious diseases; however, for fish, no pathognomonic signs are described and more than one aetiology needs to be investigated simultaneously to provide a correct and fast pathogen identification. In this respect, multiplex PCR can provide an efficient method to discriminate quickly among fish pathogens or genetic variants associated with similar clinical signs [4,5].

Real-time PCR is another widely applied PCR-based method able not only to detect the target DNA but also to quantify the number of DNA molecules initially present in the sample. For this reason, the method is also known as quantitative PCR (qPCR) [6]. In real-time PCR, the amplified product is monitored through the fluorescence of dyes or probes introduced into the reaction, eliminating post-PCR manipulations. For this reason, real-time PCR allows for processing a large number of samples with a lower level of contamination, making it more suitable for routine diagnostics compared to conventional PCR. On the other hand, a conventional PCR can be used to produce amplicons for sequencing and phylogenetic analyses [1]. In addition to diagnostics, real-time PCR can be very useful for virus detection and quantification in pathogenesis studies, especially for viruses with low or no cultivability [7].

More recently, digital PCR is taking hold thanks to the recent development of new instruments and chemistry, which have made it a much simpler and more practical technique [8]. This innovative approach has been recently applied for the diagnosis of carp edema virus (CEV), a pathogen that may lead to high mortality in both koi and common carp populations and for which no effective control methods have been developed. In this case, a prompt and efficient detection of the virus is the only approach for the prevention of further spread of the disease. In this regard, the developed digital PCR assay provides a robust diagnostic tool for the sensitive detection of CEV [9].

Despite their unparalleled contribution, PCR-based techniques also have some limits. In particular, they require expensive reagents and equipment. Isothermal amplification methods overcome this shortcoming as they are performed at one reaction temperature under simple conditions and thus do not rely on specialised equipment [10]. Among the several isothermal amplification techniques described so far, loop-mediated isothermal amplification (LAMP) is one of the most popular, with assays developed for the detection of several fish and shellfish viruses. More advanced and complex methods, such as the systematic evolution of ligands by exponential enrichment (SELEX), have been applied so far to a more limited number of fish viruses. This method was used to generate aptamers with high specificity and affinity for Singapore grouper iridovirus (SGIV)-infected cells. These aptamers can find several applications in both disease diagnosis and control; indeed, they can be used as molecular probes to set up rapid diagnostic assays and study the mechanism of virus infection as well as to develop antiviral treatments against SGIV infection in aquaculture [11]. In situ hybridisation (ISH) is another molecular method used for diagnostic purposes but above all to study the pathogenetic mechanisms of infectious diseases. ISH consists of a family of sensitive methods able to target specific nucleic acid sequences at the cellular level and potentially it can be used to correlate the presence of viruses to lesions [12,13]. The first in situ detection methods were characterised by the use of very sensitive radio labelled probes, but also by disadvantages as the difficulty in obtaining a precise localisation of the signals and the handling of radioisotopes. Hence, in the last few decades, several adjustments have been made in order to achieve greater sensitivity, practicability and safety. At present, non-radioactive methods such as chromogenic (CISH) and fluorescent (FISH) in situ hybridisation are used [13]. Moreover, if designed properly, these methods are also able to discriminate between the replicative and non-replicative status of a pathogen, by using specific sense or antisense probes [12,14]. Several ISH assays have successfully been developed in order to localise the presence of viruses in the tissues of aquatic animals. For instance, this approach is essential to distinguish true ostreid herpesvirus 1 (OsHV-1) active infection from passive contamination of the host, especially when the virus is detected in new host species and their role needs to be investigated [15,16].

The analysis of sequences has significantly contributed to gaining fundamental epidemiological information on viral diseases. The combination of the epidemiological approach and molecular methods has resulted in molecular epidemiology studies that help to understand disease distribution in populations and the relationship between viral strains and disease outbreaks. Sequence analysis has furthered the capacity to investigate viral evolutionary history through phylogenetic studies [17]. The genetic characterisation of novel North American marine viral haemorrhagic septicaemia virus (VHSV) strains and their comparison with traditional European freshwater strains have provided a remarkable insight into the origins of viral haemorrhagic septicaemia (VHS) in aquaculture. VHS was traditionally thought to be a disease exclusive to freshwater rainbow trout farming. North American and European strains belong to different genotypes that were indicated to have become separated a long time before fish farming was established on both continents. This finding has led to several new studies, which in the end proved the existence of long-standing natural marine reservoirs of the VHS virus [17].

At last, the advent of next generation sequencing (NGS) has greatly contributed to the discovery of emerging pathogens in the fish farming industry. The reduction of sequencing costs and the availability of efficient computational resources have led to a wider use of large-scale DNA/RNA sequencing. In particular, the research on unculturable and emerging viruses benefited above all by metagenomics and other methods that are not dependent on laboratory propagation [18]. The NGS approach unveils novel and often surprising findings for all viruses investigated, including the reassignment to the correct taxonomic family of an entire group of viruses such as the sturgeon nucleocytoplasmic large DNA viruses (NCLDVs), a group of viruses historically known, but mistakenly considered, as iridoviruses [19].

This review aims to illustrate how molecular methods contribute to gaining crucial knowledge about viral aquatic animal diseases reporting typical applications to some of the most challenging fish viruses. In particular, two groups of large dsDNA viruses, such as the nucleocytoplasmic large DNA viruses and the herpesvirales group, were chosen. Molecular methods have constituted a powerful tool to gain crucial knowledge on the large and complex genomes that characterise these viruses, overcoming limitations due to poor in vitro cultivability, strict species-specificity and different evolution dynamics. On the other hand, an example of a group of small RNA viruses is also reported: the nodaviruses. The research on this fast-evolving group of viruses greatly benefited from the application of molecular methods as well, providing successful diagnostic methods and allowing the exploration of the relationship between genes and biological characteristics.

## 2. Nucleocytoplasmic Large DNA Virus

The nucleocytoplasmic large DNA viruses (NCLDVs) are double-stranded DNA viruses of eukaryotes with a monophyletic origin [20]. This group includes several virus families, such as Poxviridae, Asfarviridae, Iridoviridae, Phycodnaviridae, Marseilleviridae, Ascoviridae and Mimiviridae. The sequencing of several viral genomes in the last two decades has substantially advanced knowledge of the evolutionary history of these viruses, and now all these families are officially joined in the phylum Nucleocytoviricota within the virus kingdom Bamfordvirae of the realm Varidnaviria by the International Committee on Taxonomy of Viruses (ICTV) (https://ictv.global/taxonomy accessed on 2 February 2023).

### 2.1. Iridoviridae

The family *Iridoviridae* comprises several viruses infecting fish, included in the subfamily *Alphairidovirinae* and grouped in three genera: *Lymphocystivirus*, *Megalocytivirus* and *Ranavirus* [21]. Lymphocystivirus is a group of viruses historically known and associated to a cutaneous proliferative benign disease of demersal fish since its first isolation in cell culture in 1962 [22]. Megalocytiviruses and ranaviruses have been reported long after in the 1990s and are responsible for systemic infections leading to high mortality outbreaks in cold-blooded vertebrates [23,24].

Iridoviruses possess large, linear, double-stranded DNA genomes that are circularly permuted and terminally redundant [25]. Virions have an icosahedral symmetry and infectious particles may be either “naked” or enveloped depending on their release by cell lysis or by budding from the plasma membrane, respectively [24].

PCR-based methods and phylogenetic analysis have been applied to the study of all vertebrate iridoviruses implementing diagnostic capacity, epidemiology knowledge including geographical distribution, host range and their taxonomy. Moreover, whole genome sequencing has permitted further exploration of their genome characteristics, bringing to attention interesting data on their genomic organisation and differences.

#### 2.1.1. Lymphocystivirus

Lymphocystiviruses are responsible for lymphocystis, a self-limiting disease of teleosts known since the 19th century. Lymphocystis typically presents with cutaneous nodular masses consisting of hypertrophied fibroblasts.

On the basis of typical nodular masses on the skin and fins, lymphocystis has been described in more than 140 wild freshwater and marine water fish across the globe [26]; however, the actual identification of the virus has been conducted in a more limited number of cases. Electron microscopy can confirm the presence of icosahedral iridovirus-like virions inside hypertrophied fibroblasts, and this method has been used for a long time to identify the virus in pathological tissue [26]. However, this approach limited the identification, at most, to the family level, resulting in a considerable degree of uncertainty. When partial/complete genetic characterisation was conducted, considerable differences were reported among lymphocystiviruses, despite the consistent clinical presentation described across the affected species.

Thanks to whole genome sequencing and phylogenetic analysis, four species of LCDV were recognised and named LCDV1-4. LCDV1 is reported in flatfishes (family Pleuronectidae), LCDV2 is isolated from olive flounder (*Paralichthys olivaceus*), LCDV3 is found in gilthead sea bream (*Sparus aurata*), and LCDV4 is detected in the whitemouth croaker (*Micropogonias furnieri*) [21,27]. Significant discrepancies have been pointed out in the genome size, whole genomic structure and organisation of different LCDV species [28]. In particular, LCDV1 differs significantly from other LCDVs with a genome of about half the size (1000 Kbp) compared to other LCDV species (180–203 Kbp). Comparison between the genomes of different LCDV showed a low percentage of nucleotide identities, indicating the almost complete genomic dissimilarities and non-relatedness. In particular, only some LCDV1 genomic regions were found in LCDV2-4, but the spatial arrangement of these regions was different [28]. On the other hand, viruses within a species showed a similar genome size and similar conserved genomic regions as demonstrated for viruses belonging to LCDV1 [28], at least in the case where more than one virus per species was fully sequenced.

The family *Iridoviridae* belongs to the NCLDVs, whose genome is characterised by a small number of widely shared genes and unique or species-specific operating reading frames (ORFs). These distinctive genes are possibly acquired by gene duplications, deletions, lateral gene transfers from their hosts and the de novo creation of protein-coding genes; more strikingly, it is increasingly understood that these mechanisms are driven by environmental factors [29]. Even if not fully demonstrated, differences in LCDV genomes may be due to these mechanisms of rearrangement induced by the environment in which they evolved; in fact, all LCDV have a common lifestyle, having been detected all from demersal fish [28].

**Figure 1:**
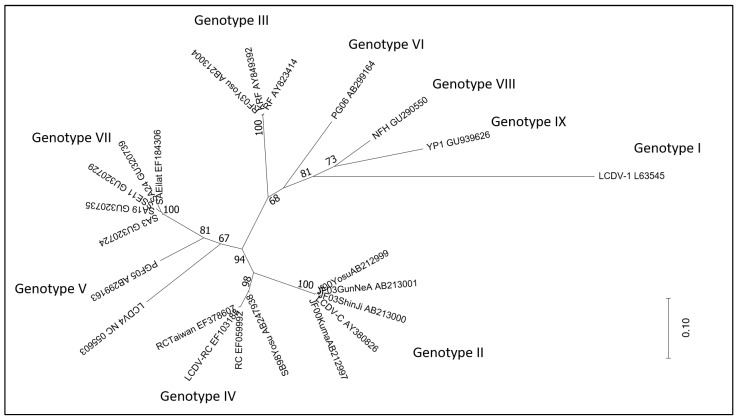
Maximum likelihood phylogenetic tree (MEGA 6 software) constructed with the major capsid protein (MCP) gene of LCDVs detected in several fish species. Strains are reported with the isolate name and GenBank accession number. Bootstrap values (1000 replicates) above 65% are shown. Substitutions per site are reflected by branch lengths (scale at bottom right).

The use of the major capsid protein (MCP) gene, a more limited area of the genome, allowed us to include in the analysis a broader number of LCDV detected in a wide range of fish host species. The MCP gene, which is characterised by extensive conservation, is employed in iridovirus comparative phylogenetic research. Sequence comparison of the MCP genes of several lymphocystiviruses clusters LCDVs into nine genotypes [26]. LCDV4 was recently added to them [27]. Full genome sequencing clearly distinguishes LCDV4 as a separate viral species; accordingly, analysis of the MCP gene segregates it separately from all other known genotypes, despite this being supported by a quite low bootstrap value (Figure 1). The size of the sequence and the genes used in the phylogenetic analysis can have a significant impact on the output and its robustness.

A phylogenetic analysis of a number of MCP gene nucleotide sequences showed that most of the LCDV were grouped by similar hosts [28].

Through experimental trials, species-specificity has been confirmed for some genotypes. Indeed, olive flounder and rockfish (*Sebastes schlegeli*) isolates were experimentally infected by their respective homologous isolates but not by the heterologous isolates [30]. However, MCP gene sequencing revealed that the majority of the genotypes are species-specific or have a limited range of host while some exceptions were identified. Gilthead sea bream and Senegalese sole (*Solea senegalensis*) isolates showed very high MCP nucleotide similarity and clustered in the same genogroup by phylogenetic analysis [31].

Benkaroun and colleagues [28] suggest that the virus speciation could be possibly due to different geographic locations. The observation that the LCDV sequences characterised from three different wild fish species, with similar behaviours and habitats, and that are commonly found in the North Sea, grouped together support this.

Nevertheless, even in this case, some exceptions have been described. High similarity has been observed among sequences of LCDV found in different geographical areas. In some cases, it can be easily explained due to the commercial movement of fish, whereas in other circumstances it is more difficult to explain, such as in the case of an LCDV isolate from a grey gurnard (*Eutrigla gurnardus*) from the North Sea that clustered with an LCDV isolate from a tropical ornamental fish species from South Korea [28].

Moreover, the sequencing and phylogenetic analysis of a 306-bp fragment of the MCP gene of an isolate detected in juvenile gilthead seabream, imported from a Mediterranean hatchery to a farm in Egypt, demonstrated the presence of LCDV1, originally associated with lymphocystis disease in Northern European countries, expanding de facto both the host and the geographical range of this virus species [32].

Molecular methods also contribute to advancing the knowledge on lymphocystis pathogenesis. The LCDVs show tropism for the dermal tissues infecting fibroblast cells that typically appear enlarged with inclusions and a central nucleus. PCR-based investigations also showed the presence of the virus in many other tissues, including internal organs such as the brain and spleen, pointing out a systemic infection in several species affected by lymphocystis [7,33,34,35]. A further study, applying the ISH technique along with immunohistochemistry (ISH) to experimentally infected gilthead seabream, showed the involvement of hepatocytes and macrophages as target cells for virus replication in addition to the fibroblasts [36]. The implementation of real-time PCR methods, specific for a fragment of the LCDV MCP gene, further improved the diagnostic and study abilities. These methods, in fact, showed a high level of sensitivity and were successfully applied to detect LCDV directly from asymptomatic fish tissues. For this reason, this method can be applied to screen carrier fish [7,37]. The ability of this method not only to detect but also to quantify LCDV in both diseases and asymptomatic fish showed its suitability for pathogenesis studies [7].

To further make the LCDV diagnosis faster and easier, LAMP assays were developed to detect two different lymphocystiviruses. LAMP requires a set of several specific primers that recognise different sequences on the target DNA, achieving highly selective nucleic acid amplification. Due to its high specificity, two different assays were developed, one for the LCDV2, infecting the olive flounder [38] and one for the LCDV3 (also known as genotype VII), which is widespread in gilthead seabream populations [39]. Both the assays provide a fast, reliable, sensitive and specific diagnostic method that can be used for routine diagnosis and surveillance programs in well-equipped laboratories as well as under field conditions.

#### 2.1.2. *Megalocytivirus*

The implication of iridoviruses other than lymphocystivirus in fish pathology has further stressed the importance of using reliable and detailed (in-depth) identification methods able to distinguish different viruses that can show a similar morphology (irido-like) and cause similar effects on tissues, such as cell enlargement.

Viruses within the genus *Megalocytivirus* are causative agents of severe disease accompanied by high mortality in multiple species of marine and freshwater fish. Hitherto, megalocytivirus infection has been reported in more than 50 species of fish, resulting in epidemics in ornamental fish, fish farmed for food and also wild fish [1,23].

Sequence analysis clearly segregates megalocytivirus as a separate cluster of viruses with respect to other fish iridoviruses [23]. Adenosine triphosphatase (ATPase) and MCP are key viral genes for megalocytivirus phylogenetic analysis. In fact, most megalocytiviruses show >94% sequence identity within these genes, whereas sequence identity with ranaviruses and lymphocystiviruses is <50% [21]. Furthermore, phylogenetic analyses of these genes cluster megalocytiviruses into three groups: the red seabream iridovirus (RSIV), the infectious spleen and kidney necrosis (ISKNV) and the turbot reddish body iridovirus (TRBIV).

The red seabream iridovirus (RSIV) subgroup comprises megalocytivirus typically isolated from red seabream (*Pagrus major*) and other marine fish species; the infectious spleen and kidney necrosis (ISKNV) subgroup includes most of the megalocytivirus detected in ornamental fish species, primarily from freshwater fish and the turbot reddish body iridovirus (TRBIV) has been detected mainly from flatfishes, such as flounder and turbot, although some isolates have also been reported from barred knifejaw (*Oplegnathus fasciatus*) and from ornamental fish species [23,40].

Moreover, further viruses clustering with *Megalocytivirus* but forming separate branches within this genus have been reported. The threespine stickleback iridovirus (TSIV) is the first megalocytivirus isolated from a wild temperate North American fish, the threespine stickleback (*Gasterosteus aculeatus*) [41]. The scale drop disease virus (SDDV) was isolated from Asian seabass (*Lates calcarifer*) affected by the scale drop syndrome, a severe illness with, until then, an unknown aetiology causing significant economic losses in Asian seabass since its first description in 1992 [42]. The European chub iridovirus (ECI) was another divergent megalocytivirus isolated from moribund European chub (*Squalius cephalus*) [43]. Sequences obtained from traditional Sanger sequencing as well as from next-generation sequencing have been fundamental in the characterisation of these viruses and in their correct taxonomic placement. Particularly, the application of the virus discovery cDNA-AFLP (VIDISCA) approach played a fundamental role in the discovery of the scale drop disease virus [42]. This method consists of a library preparation method that has been successfully used to identify several novel viruses [44,45,46]. The next generation sequencing of the VIDISCA library of sera from fish affected by scale drop syndrome pointed out the sequences of an unknown virus, which was named scale drop disease virus. Starting from the sequences obtained by the VIDISCA approach, a real time qPCR was developed and the near complete genome sequence of the novel virus was obtained via genome walking. The correct identification of the aetiological agent was the starting point to develop efficient diagnostic and control methods [42].

The application of PCR-based methods, genetic and phylogenetic analyses was also very useful to investigate the distribution of already-known megalocytiviruses.

The first outbreak associated with a megalocytivirus was recorded in cultured red sea bream in Japan in 1990 and the virus was designated red sea bream iridovirus disease (RSIVD) [47]. A genetic investigation on archival ornamental fish samples, on the other hand, dated the megalocytivirus index case back to 1986 [40]. Furthermore, this study shows how the application of molecular techniques to archival formalin-fixed and paraffin-embedded (FFPE) materials can provide valuable taxonomic information relating to historical accounts of megalocytivirus-like infections in ornamental fish [40]. The sequence analysis of megalocytivirus cases in ornamental fish observed over an extended period of time showed an interesting distribution of different megalocytivirus species in this group of fish. The TRBIV genotype prevailed among megalocytiviruses affecting ornamental fish since the late 1980s and until the early 1990s, whereas after this date the ISKNV subgroup became the megalocytiviruses most frequently detected in ornamental fish [40].

**Figure 2:**
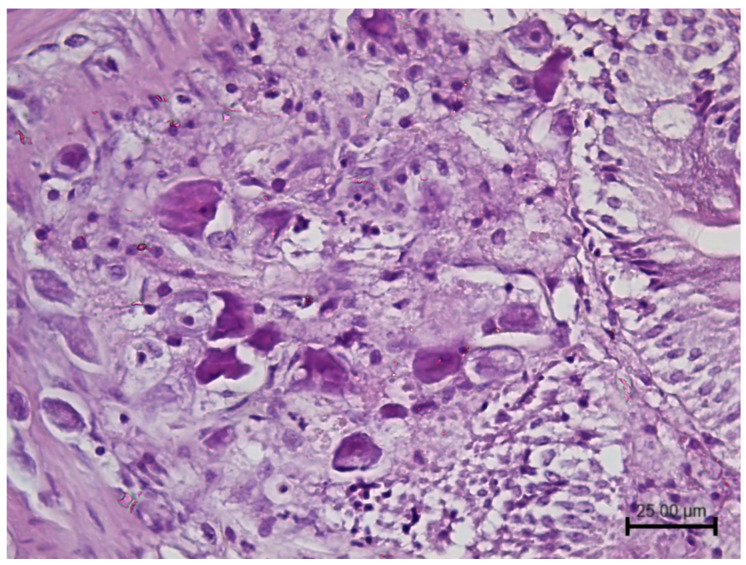
Histopathology of intestine from a megalocytivirus-infected prickly leather-jacket (*Chaetodermis penicilligerus*) showing multiple hypertrophic cells containing granular basophilic viral inclusions. Haematoxylin and eosin (H&E) stain. (Courtesy Prof. Gian Enrico Magi University of Camerino, Italy).

Megalocytivirus infections are associated with severe systemic diseases. Histologically, enlarged, basophilic, inclusion body-bearing cells (IBC) can be observed within infected organs. IBC are made up of hypertrophied cells with large foamy or granular basophilic inclusions that enlarge the cytoplasm and displace the nucleus (Figure 2).

IBC cells are considered pathognomonic for infection with megalocytiviruses; however, histology is not conclusive. IBC has been misinterpreted as amoebae, and other non-iridoviral viruses have been associated with some cases [40,48]. Furthermore, the presence of more than one viral species and several genotypes requires diagnostic methods able to distinguish among them. Being the RSIVD listed by the World Organisation for Animal Health (WOAH), it is critical to identify the correct megalocytivirus species to monitor and report it correctly [49]. For this purpose, the WOAH describes diagnostic PCRs that enable the differentiation of RSIV from ISKNV by sequencing or by an RSIV-specific PCR [50]. Moreover, universal and subgroup-specific PCR assays have been set up to amplify all megalocityviruses or only those belonging to a subgroup (RSIV, ISKNV and TRBIV), respectively [23]. In addition to a conventional PCR, a real-time PCR approach has also been applied to megalocytivirus to set up a rapid, specific and sensitive detection method to facilitate surveys of apparently healthy ornamental fish, especially for those subjected to movement, representing a risk of virus spreading across countries [51].

In addition to several PCR assays, the isothermal amplification approach has also been used to develop efficient diagnostic methods for megalocytivirus. A LAMP assay has been developed for each megalocytivirus subgroup: RSIV, ISKN and TRBIV. All three assays showed a sensitivity greater than a conventional PCR and the ability to detect the virus without the use of gel electrophoresis, making these assays suitable for rapid field diagnosis [52,53,54]. A similar approach has been used to set up a rapid field diagnostic method to detect the SDDV. This assay combined the clustered regularly interspaced short palindromic repeats-associated protein 12a (CRISPR-Cas12a)-based nucleic acid detection technology with recombinase polymerase amplification (RPA), reaching high sensitivity and accuracy and the ability to rapidly detect the virus in asymptomatic fish [55].

On the whole, the contribution of the molecular methods has been and continues to be invaluable in the diagnosis and study of megalocytiviruses and throughout the *Iridoviridae*. However, proper data handling and interpretation are required to avoid misunderstandings.

As above mentioned, MCP and ATPase are key genes in megalocytivirus phylogenesis and their analysis generally provides comparable results. However, genetic analysis also showed potential exceptions to this concordance, such as the megalocytivirus detected in African lampeyes (*Lacustricola centralis*). African lampeye iridovirus (ALIV) clusters with the red seabream-like megalocytivirus on the basis of the sequence of the ATPase gene, whereas phylogenetic analysis of the MCP gene sequence places this virus firmly as a member of the ISKNV subgroup. This result could be due to the presence of both ISKNV and RSIV-like viruses in African lampeye samples; alternatively, ALIV could be a chimeric virus displaying the MCP of ISKNV and the ATPase of RSIV, so further research is required to resolve this question [23,56].

### 2.2. Sturgeon NCLDVs

NCLDVs have also been detected in sturgeons; however, so far, sturgeon NCLDVs have not been officially classified as belonging to any of the virus families in this group. Sturgeon NCLDVs are increasingly widespread in sturgeon farming in North America and Europe, causing severe losses to the sector and therefore eliciting more and more interest. However, they have never been isolated in cell culture and this has significantly limited their study and detection. The identification of this viral infection in sturgeons has been historically made through histology and electron microscopy to visualise typical microscopic changes, consisting in prominent enlargement of the infected cells containing an abundant, homogeneous, amphophilic to basophilic cytoplasm and viral particles, respectively [57]. On the basis of the morphology of the virions, these viruses have long been reported as irido-like; however, sequence analyses unveiled a different scenario.

Due to their uncultivability, molecular methods have been the only essential but challenging tool to study these viruses. NCLDVs have been reported in nine sturgeon species of genera *Acipenser*, *Scaphirhynchus* and *Huso*, with significant sequence differences among the viral strains detected in different host species leading to the failure of PCR protocols developed on a different strain [58]. However, the design of degenerate primers on a vast alignment of MCP sequences, including several different strains, led to a PCR protocol able to detect several different sturgeon NCLDVs, including both European and American strains [59].

The phylogenesis of the NCLDVs is particularly difficult due to the fact that their genome evolution involved the lineage-specific expansion of some genes but also the acquisition of numerous genes via horizontal gene transfer from the eukaryotic hosts, other viruses and bacteria [20]. Therefore, the choice of the correct gene/genes for phylogenetic study is crucial. A reliable method for the taxonomic classification of NCLDVs to the species, genus and family levels is the phylogenetic analysis of MCP gene sequences [25].

Phylogenetic analyses of NCLDVs, including North American sturgeon viruses and members of the families *Mimiviridae*, *Phycodnaviridae*, *Iridoviridae*, *Marseilleviridae*, *Ascoviridae* and *Asfarviridae*, using the MCP gene, showed that sturgeon NCLDVs form a cohesive taxonomic group with a distinct evolutionary lineage within the class *Megaviricetes* to which all these viruses belong. Furthermore, this study shows that, on the basis of phylogenetic analyses using the MCP gene, the sturgeon NCLDVs could be identified to the species or possibly sub-species level. Sturgeon NCLDVs collected from different river basins in North America form distinct taxonomic units located at the tips of the MCP-based phylogenetic tree, defining different North American genotypes. Regarding host specificity, the virus MCP genotypes were not exclusively associated with host species, but MCP analysis suggests that they may be host family- or genus-specific [60]. Analysis of European NCLDVs based on the same gene showed that they form a homogeneous cluster that is closely but distinctively related to North American NCLDVs [59,61].

More recently, a phylogenetic study was conducted using nucleocytoplasmic virus orthologous genes (NCVOGs). NCVOGs are a set of genes that can be mapped onto the genomes of extant NCLDVs, with five NCVOGs shared by all NCLDVs. Going down to the family level, the number of common genes increases, with some genes proposed as hallmark features for a specific family that, in the case of *Mimiviridae*, have been named MimiCOGs. The application of next-generation sequencing to tissues collected from lake sturgeons associated with Namo virus (NV) mortality outbreaks led to the determination of a novel 306,448 bp long genome sequence, including orthologous genes. Nine orthologous protein sequences were specifically found by analysing this larger portion of the NV genome, one of the North American sturgeon NCLDVs, which enabled the expansion of the phylogenetic investigation of NV beyond the MCP. These nine proteins are regarded as appropriate phylogenetic markers since they are present in all or almost all of the seven virus families of the NCLDV. Despite not being conclusive, this study suggests that NCLDVs represent a new virus lineage in the family *Mimiviridae* [19]. The *Mimiviridae* family gathers together several peculiarities, most of which were discovered owing to the use of molecular methods. *Mimiviridae* includes some of the largest known DNA viruses, both in terms of particle size and genome complexity [62]. The Tupanvirus deep ocean genome consists of 1.51 Mbp, encoding 1425 predicted proteins, surpassing the number of genes encoded by many bacteria and by the smallest parasitic eukaryotic microorganisms [63,64]. However, *Mimiviridae* is a very large and diverse family of eukaryotic viruses, with the majority is isolated from aquatic environments, including fresh, brackish, and seawater environments. This family is known to have a vastly variable host range, infecting eukaryotic unicellular organisms from at least five major phyla, including Amoebozoa, Haptophyta, Chlorophyta, Excavata and Heterokonta. If NV is confirmed to be a member of the *Mimiviridae*, this family will have the broadest host range of any known aquatic virus, ranging from algae to fish [64].

## 3. *Herpesvirales*

The order *Herpesvirales* includes numerous viruses with a similar structure, genome and biological properties. This order comprises three phylogenetically related families that infect a wide range of hosts. Among these, the *Herpesviridae* family, which includes viruses able to infect mammals, birds or reptiles, is, by far, the most important, in terms of both the number of the viral members and the studies that have been carried out on it. The *Malacoherpesviridae* family encompasses viruses that infect molluscs. Finally, the *Alloherpesviridae* family, to which an increasing number of studies have been devoted over the last decades, includes viruses that infect fish and amphibians [65].

Aside some differences related to host specificity, tissue tropism, replication kinetics and pathogenic potential, all herpesviruses (HVs) have a common morphology and the ability to establish a persistent infection.

The HVs’ virion structures are remarkable conserved. The genome, consisting of double-stranded DNA, is densely packed as a core surrounded by an icosahedral capsid [66]. The nucleocapsid, is embedded in the tegument, an irregular layer of globular material externally surrounded by the envelope, a lipid bilayer membrane where several glycoproteins are anchored [66,67].

The analysis of viral genomes suggests that herpesviruses have diverged from a common ancestor through co-speciation with their host species via species-specific latent infections [68]. Mostly, these viruses are well-adapted to a single host species as a result of long-term coevolution [67]. As a matter of fact, the presence of viral genes originated from the host genome confirms the strict co-evolution of these viruses and the reservoir species to the extent that some genome traits seem to be more similar to the host genome than to the genome of viruses belonging to the same family [67].

Indeed, herpesviruses are common host-specific pathogens that can cause a variety of diseases, ranging from asymptomatic infections to mild, moderate or severe disease, depending on the intrinsic traits of the host and biological features of the virus. Under specific conditions, including co-infection, a weak immune system, an abnormal or naive host, environmental circumstances, and/or distinct pathogenic virus strains, HVs can be highly pathogenic [69]. They have proven to be difficult to isolate in cell culture due to their species-specific nature. The ability to detect these viruses using transmission electron microscopy (TEM) has been demonstrated to be effective and this method has been used to investigate a number of suspected disease-causing herpesviruses in fish without propagating the virus in cell culture [70]. These viruses are often detected by ultrastructural investigations and are morphologically described, whereas they are, so far, less genetically characterised, except in some cases [71,72,73].

The implementation of new powerful molecular methods in recent years has allowed for better study of herpesvirus whole genome sequences and detailed phylogenetic analyses [66]. Within the *Alloherpesviridae* family, for example, the discovery of several conserved genes has allowed the establishment of degenerate primers, used in PCR-based methods to identify the phylogenetic relationship among the viruses of this family, also allowing the characterisation of unculturable herpesviruses such as the SalHV3 [66,74].

Furthermore, by the application of molecular tools, it was possible to establish the genetic diversity of viral strains that are spread in different geographical areas, as demonstrated for CyHV-3 in Europe [66].

The study of viral genomes is also important to develop adequate instruments to control the diseases. In fact, the scientific advances in molecular biology and molecular virology paved the way for the development of an attenuated recombinant vaccine against CyHV-3. Particularly, using new rational design approaches, the risk of reversion to virulence of a live attenuated viral vaccine can be avoided by editing the viral genome and deleting the genes that encode for virulence factors [65].

### 3.1. Alloherpesviridae

The family *Alloherpesviridae* contains four genera: *Batrachovirus,* which comprises viruses responsible of amphibian diseases, and three genera, that cause diseases in fish, named *Cyprinivirus*, *Ictalurivirus* and *Salmonivirus* [75]. To date, all the characterised alloherpesviruses appear to cause disease in only one species of fish or in closely related members of the same genus [66].

#### 3.1.1. *Cyprinivirus*

In the genus *Cyprinivirus*, there are three Cyprinid herpesviruses: the cyprinid herpesvirus-1 (CyHV-1) that was isolated in Japan from a papillomatous skin growth on an infected koi carp (*Cyprinus carpio*), the cyprinid herpesvirus-2 (CyHV-2) that primarily affects goldfish (*Carassius auratus*), causing severe hematopoietic necrosis and CyHV-3, identified as the causative agent for mass mortality outbreaks in koi carp [76].

It has been demonstrated that CyHV-1 is the aetiological agent of carp pox, a disease affecting carp and other cyprinids and characterised by the presence of flat epidermal papillomas. Normally, these lesions are seasonal and tend to disappear naturally when the water temperature rises above 20 °C. Ultrastructural investigations of carp pox have documented the presence of viral particles morphologically similar to herpesviruses [77,78]. Isolation of the virus from the papillomatous tissue in cell culture using cell lines such as Fathead Minnow (FMH-1) or Koi Fin (KF-1) cell lines is difficult because the appearance of the infectious virus depends on the developmental stage of the papillomas. An ISH assay using a DNA probe has also been developed for research purposes [79,80,81]. Apart from the development of these methods in the 1990s, relatively few advancements in research were made. Infections associated with CyHV-1 have been known for decades and are easily recognised by the presence of recurrent benign epidermal proliferations. However, it has been a neglected disease for a long time due to the limited tools suitable for its aetiology identification and study. In this respect, the application of molecular methods has fostered the research allowing to investigate some pathogenetic aspects of the disease. Particularly, with the advent of molecular techniques and the possibility to employ at the same time several primers, multiplex and broad-range PCR assays have been developed and used together with DNA array hybridisation to detect and concurrently identify all the cyprinid herpesviruses (CyHV-1, CyHV-2 and CyHV-3), as well as some of the most important fish pathogenic Flavobacterium species [82]. However, apart from these research papers, the understanding of the pathogenesis of this disease is far from being known, and currently there are no reports concerning the detection of CyHV-1 within the proliferated epidermal cells by using in situ hybridisation techniques. Recently, the presence of CyHV-1 has also been reported in association with the malignant counterpart of the epidermal neoplasm, the squamous cell carcinoma. However, the presence of a viral active replication in pathological tissue was ruled out by RT-PCR analysis targeting the viral mRNA [83]. The use of RT-PCR for the research of transcripts is useful to study the DNA virus and determining whether or not they replicate; this approach has also been used to investigate the presence of viral replication in other fish DNA viruses [39,84].

CyHV-2 is an emerging viral pathogen that infects several fish from the genus *Carassius* (i.e., *Carassius auratus*, *Carassus gibelio* and *Carassius carassius*) [85,86,87]. Its genome is composed of a large double-stranded DNA. The virus is responsible for herpesviral hematopoietic necrosis (HVHN), a disease characterised by acute gill haemorrhage that can lead to high mortality rates [88]. Surviving individuals can become chronic carriers and spread the pathogen without displaying clinical signs [89].

Due to the tendency of the virus to spread globally, disease control has become a worldwide priority and the understanding of the infection mechanisms coupled with the development of an efficient diagnosis plays a key role [90].

The in vitro isolation of CyHV-2 has been very challenging due to the difficulty of growing the virus on permissive cell lines [66]. The available cell lines, such as Epitelioma papulosum cyprinid cells (EPC), FHM and KF-1 are able to successfully subculture the virus only five times [91]. However, recently, the gold fish cell line (GFF), gibel carp cells (GiCB) and the gibel carp caudal fin cell line (GiCF) have proven to be highly permissive for the isolation and propagation of CyHV-2 [91,92,93].

Electron microscopy (EM) has also been used to detect the presence of herpes-like virions in tissues of CyHV-2-suspected fish. Nevertheless, EM is not conclusive in identifying CyHV-2 and furthermore, it is highly technical and time-consuming, making it unable to be used in routinary diagnosis [90].

Therefore, the diagnostic confirmation of the presence of CyHV-2 is still strongly based on the application of molecular methods. PCR protocols able to detect conserved regions of DNA polymerase and the major capsid protein (MCP) genes of CyHV-1, CyHV-2 and CyHV-3 have been developed [94].

Furthermore, specific PCR diagnostic methods for CyHV-2 detection were also established. For example, Goodwin and colleagues [95] developed a real time 5′nuclease PCR method (Taqman) to quantitatively detect CyHV-2. The method resulted specifically for the detection of this virus in clinical cases of HVHN as well as in apparently healthy fish and did not show cross-reactivity with the other CyHVs.

To simplify and expedite the diagnostic process, an isothermal amplification approach has also been used to develop rapid methods suitable under field conditions.

The LAMP may be considered as a useful alternative to the conventional PCR-based technique for the rapid detection of CyHV-2. The method developed by He and colleagues [96] and targeting the conservative terminase gene, resulted to be an effective, rapid and highly sensitive method for the detection of CyHV-2 without cross-reacting with other fish bacteria and viruses.

Furthermore, an RPA assay developed in combination with a lateral flow dipstick (LFD) represents a quantum leap if compared to the traditional PCR assays, due to its high sensitivity, specificity and applicability [97]. In fact, this method has proven to be useful for the rapid detection of several fish viruses, including CyHV-2, using simple equipment and showing its suitability for the in-field application [97,98].

Moreover, it is worthwhile to note that molecular methods, such as complete genome sequencing, comparative genomics and molecular characterisation have gained recent interest in further exploring the genome structure and the potential molecular and pathogenic mechanisms of CyHV-2 [99]. In fact, even if the genome of CyHV-2 has been completely sequenced, the molecular pathogenesis of the virus remains poorly understood. As with other DNA viruses, the genes of CyHV-2 are expressed in three temporal phases: immediate-early (IE), early (E) and late (L) genes. The setup of specific molecular methods could be able to detect these gene specifically and acquire important information for viral molecular characterisation [88].

However, PCR-based methods as well as viral in vitro isolation are not able to determine if a pathogen is unequivocally responsible for clinical signs or pathogenic lesions. Conversely, in situ hybridisation techniques combining the precision of molecular genetic information with microscope visualisation are potentially able to correlate viral pathogens to tissue alterations. Ding and colleagues [90] developed an oligonucleotide probe-based fluorescent in situ hybridisation technique, able to detect the presence of CyHV-2 in the tissues of infected animals. The designed probes used in the study did not hybridise with the tissue of the uninfected fish host and did not cross react with other tested viruses (i.e., channel fish virus-CCV; grass carp haemorrhagic virus-GCHV; infectious spleen and kidney necrosis virus-ISKNV), demonstrating that they are a specific and powerful tool for the detection of CyHV-2 in host tissues [90].

CyHV-3 is also known as koi herpesvirus (KHV), and over the last years there has been an enormous increase in the knowledge of KHV, koi herpesvirus disease (KHVD), its pathogenesis and virus variants thanks to the application of several molecular methods. Isolation on cell cultures is considered the gold standard for the diagnosis of several viruses including those infecting fish. However, this approach has a limited application in KHV diagnosis. The KF-1 cell line was developed to isolate KHV; however, cell culture is time-consuming and overall it is less sensitive than PCR for KHV detection [100]. On the whole, KHV can be isolated in only a limited number of cell lines, which can be difficult to handle. On the other hand, molecular methods are considered the best approach for KHV diagnosis. Notably, it is recommended that a presumptive diagnosis of KHV disease should be made on the basis of clinical signs and pathology, with confirmation gained using CyHV-3 PCR [101]. Considering that viral isolation has shown its critical issues, identifying a molecular method that is as specific as possible has taken a long time; CyHV-3 has also entailed an intensive interlaboratory comparison, mainly related to the need to identify surveillance methods. At present, PCR-based assays are the primary method used to detect KHV-infected fish. Several PCR methods using the thymidine kinase [102] and sph1 genes [103,104] are currently recommended in the WOAH’s *Manual of Diagnostic Tests for Aquatic Animals* as well as in Japanese guidelines for KHV diagnosis [105]. Furthermore, PCR, followed by sequence analysis of the PCR products is also considered the most reliable method for confirmatory identification of a virus that has caused a cytopathic effect in cell cultures [106]. In order to operate viral surveillance, Yuasa and colleagues [107] developed highly effective *intra vitam* assays to detect KHV in targeted fish; particularly, in this study, the efficiency of four *intra vitam* assays in detecting KHV in koi carp was compared on each day after the experimental exposure to the virus. The obtained results showed that PCR assays conducted on the gills and scales are able to detect KHV for apparently longer periods than the other assays. Furthermore, the study also suggests the application of the PCR method to environmental samples as a convenient *intra vitam* assay.

Concerning the use of more complex methods, the scientists had increasingly felt the need to develop a multiplex PCR, to obtain a rapid result about the presence of multiple pathogens. Multiplex PCR assays have grown in popularity in recent years due to their low cost and time requirements, as well as their ability to detect multiple pathogens at the same time. Notably, both conventional and real-time PCRs have effectively been multiplexed to differentiate the genetic variants of fish pathogens. Generally, real-time PCR assays is not only more sensitive than conventional PCRs and able to provide quantitative information but, eliminating post-amplification steps, it is also able to reduce cross-contamination. However, if compared to a conventional PCR, a real-time PCR requires a higher investment in terms of cost and expertise. By contrast, a conventional PCR, despite being less performing, can be applied on a large scale to diagnostic laboratories including small laboratories supporting the aquaculture industry [4,5].

Concerning CyHV-3, a multiplex PCR was developed to make a differential diagnosis with carp oedema virus (CEV) infection, as clinical signs produced by KHV and CEV infections are overlapping. CEV and KHV are two pathogens causing concern for common carp breeders and koi fanciers worldwide. They are responsible for diseases with similar external signs, making it difficult to differentiate them clinically. In order to detect and discriminate between these two viruses, Soliman and colleagues [108] have developed and optimised rapid and accurate single- and multiplex isothermal diagnostic tools based on the RPA technology. In this study, the targeted viruses were specifically identified by the CEV- and KHV-RPA tests. The assays’ lower detection limits were comparable to those of the already established diagnostic PCR assays for the detection of CEV and KHV. Furthermore, being these assays based on an isothermal approach, they can be performed in field situations, improving the screening of fish and reducing the spread of these viruses, thereby enhancing the common carp and koi industries [108]. Using the same approach, several LAMP assays have been developed to detect quickly the CyHV-3 TK viral gene, without using a thermal cycler and with a sensitivity comparable to conventional PCR methods [76,109]. Among LAMP assays, a new fluorescence real-time LAMP method has recently been developed to simultaneously detect CyHV-3 and CEV DNA in clinical samples. The method resulted to be effective in detecting a viral presence in non-lethal mucus swabs of common carp in less than 20 min. Furthermore, it was also able to confirm a coinfection of CyHV-3 and CEV. This method presents advantageous characteristics such as good performance, cost-effectiveness and ease of use that make it suitable for in-field applications [110].

It would be extremely advantageous to use non-lethal sampling to drive decisions regarding the movement of freshwater, often associated with conservation issues also considering the practical and ethical advantages of this approach [111]. In this study, the sensitivity of seven different PCR assays able to detect KHV using lethal and nonlethal sampling methods was compared. The results obtained showed the limitations of these assays in detecting a virus during the first 4 days post infection (dpi). In particular, the time after KHV infection and the tissue sampled were linked to false-negative results. Regarding non-lethal sampling, the method appears to be effective for KHV screening, with efficient detection in mucus samples obtained from external swabs collected during the early infection period (<5 dpi), whereas gills and kidney biopsies were negative using the same PCR assays. Therefore, the reliability of KHV detection in subclinical, acutely infected carp may be increased by the use of a non-lethal sampling approach [112].

#### 3.1.2. Sturgeon Herpesviruses

Sturgeon herpesviruses are unusual in that they do not cluster by host species [75]. In particular, based on the analysis of the DNA polymerase gene, it was demonstrated that acipenserid herpesvirus 1 (AciHV-1) and acipenserid herpesvirus 2 (AciHV-2) did not cluster in the same genus. AciHV-1 is tentatively classified among the *Alloherpesviridae* family, while AciHV-2 is part of the genus *Ictalurivirus* and is akin to catfish herpesviruses [113,114]. Indeed, sequencing of approximately one half of the genome of AciHV-2 revealed that the gene organisation is very similar to that of ictalurid herpesvirus 1 (IcHV-1) [71]. Episodes of AciHV-1 disorders in white sturgeons (*Acipenser transmontanus*) have been reported since 1989. The virus is generally associated with infections of the integument and oropharyngeal mucosa and it may cause elevated losses in juveniles [115]. The AciHV-2, which was isolated a few years later after AciHV-1 had the greatest geographical and host spectrum expansion since it was reported in Europe as well as in the United States and Canada [116]. The virus has been considered a major cause of mortality among farmed juvenile white sturgeon [114].

Currently, the detection and identification of both AciHV-1 and AciHV-2 is primarily based on the use of a generic-herpesvirus PCR followed by sequencing analysis. However, the application of molecular detection to sturgeon herpesviruses is challenging. The strict virus–host co-evolution has led to the presence of similar genes in both the virus and the host. Furthermore, it is worthwhile to note that the genomes of herpesviruses are constituted by a core group of genes that control several steps in the virus cycle (e.g., viral entry into cells, DNA replication) and that these genes are often used to phylogenetically classify viruses. However, several errors have occurred in a previous study that reported the wrong designation of seven sturgeon herpesviruses in three clades [117]. Nonetheless, Kurobe and colleagues [118] revised the phylogenetic relationship among the herpesviruses isolated from sturgeons, supporting their results with the new data from the DNA polymerase and terminase gene sequences. This molecular result also agreed with the phenotypic characteristics of the cytopathic effect detected in cell cultures.

Molecular methods have also been used to study the epidemiological dynamics of sturgeon herpesviruses isolated from different geographical areas. AciHV-2 was discovered in North America in the mid-1990s and a decade later, a closely related virus was found in Europe (Russia), suggesting that the Russian isolates may have originated from North America. Moreover, Doszpoly and colleagues [116] showed that the Russian types of Siberian sturgeon herpesvirus (SbSHV 1 and 2) detected in their study have a close genetic relationship with a North American AciHV-2 strain, assuming two possible direct or indirect introductions of the virus to Russia. However, since no official direct trade between North America and Russia has occurred within the previous 15 years, they have also hypothesised a Eurasian origin of the virus and its subsequent introduction to North America.

Finally, in a paper about multifactorial causes of chronic mortality in juvenile sturgeon (*Huso huso*), NGS allowed the identification of a trace of the herpesvirus DNA polymerase (DNA pol) gene. Specific sequences were obtained and used to set up a PCR assay to analyse a larger number of samples. Unexpectedly, traces of the herpesvirus DNA pol gene were detected in both diseased and non-diseased animals, concluding that it was not related to specific mortality [119]. So, it can be reiterated that molecular methods are powerful tools but they must be well used and interpreted to avoid incorrect conclusions.

#### 3.1.3. *Ictalurivirus*

Regarding the genus *Ictalurivirus* (IcHV), two viruses responsible for substantial losses in catfish aquaculture have been so far described, the ictalurid herpesvirus 1 (IcHV-1) responsible for a severe haemorrhagic disease in channel catfish (*Ictalurus punctatus*) and the ictalurid herpesvirus 2 (IcHV-2) that causes a haemorrhagic condition in the black bullhead (*Ameiurus melas*). However, through an experimental infection, IcHV-2 was demonstrated to also be able to cause high losses in *I. punctatus* fry and juveniles [120].

The two viral species are phylogenetically close and are classified within the genus *Ictalurivirus*, with IcHV-1 being the type species [101]. Recently, the analysis of the complete IcHV-2 genome using NGS confirmed that this virus represents a distinct member of the genus *Ictalurivirus,* as already hypothesised in previous studies on the basis of the sequencing of an IcHV’s partial genome [71,113]. IcHV-1 has been the prototypic fish herpesvirus for decades. This virus was associated with high mortality rates among fry and fingerlings in the United States’ extensive catfish industry in the late 1960s. The causative agent was a virus, which showed ultrastructural features consistent with a herpesvirus. The IcHV-1 genome sequence revealed that fish herpesviruses evolved separately from mammals, birds and reptiles herpesviruses [109], establishing the basis for the current taxonomy arrangement of the order *Herpesvirales*.

Ictalurid herpesvirus 2 was first identified in reared adult black bullheads (*Ameiurus melas*) in Italy in 1994 [121]. Despite originally suspected to be an IcHV-1 outbreak associated to a long-range transmission, through sequence analysis, it was revealed to be associated to a different ictalurivirus [120].

As with other herpesviruses, IcHV-1 has been associated with latent infection, challenging the diagnosis of this condition [122]. During the latent infection, in fact, the absence of assembled viral particles can undermine the success of traditional methods such as isolation in cell cultures or antigen detection assays. On the other hand, molecular methods targeting the viral genome are effective in detecting latent viruses [123]. So, molecular methods are suggested for a confirmatory diagnosis as well as for detecting fish carriers. Several traditional PCR assays are available to detect IcHV-1. These methods are highly sensitive, so they can be applied to detect latent viruses. They have also been tested for specificity against several alloherpesviruses, but not IcHV-2. However, differentiating between the two viruses is crucial from the diagnostic point of view, since IcHV-2 has not been found in North America [120]. Since there is a similarity in the pathogenetic appearance of both IcHV-1 and IcHV-2, it is important to employ molecular tools for diagnostic purposes in order to distinguish between these two viruses.

In addition to conventional PCR, a validated IcHV-2 TaqMan qPCR based on the use of a specific hydrolysis probe has also been developed and tested against multiple isolates of IcHV-1 showing no false positive results [124].

Furthermore, the LAMP technique is an optimal method operating on a highly conserved viral IcHV genome. Hao and colleagues [125] developed a straightforward, quick and accurate IcHV-1 detection method that is crucial for early diagnosis and prompt disease control on site. The target gene ORF77, which codes for the phosphokinase protein of IcHV-1, was selected as the target region for the real-time fluorescence loop mediated isothermal amplification (RealAmp) technique developing a set of six specific primers. The reaction was highly specific as no cross-reactions to other DNA pathogens from aquatic animals, including White spot syndrome virus (WSSV), infectious hypodermal and hematopoietic necrosis virus (IHHNV), KHV, RSIV, ISKNV and SGIV were shown. The amplification curves of the parallel samples were essentially coincident, as shown by the intra- and inter-assay tests, demonstrating good reliability. These findings collectively imply that the RealAmp approach for IcHV-1 detection is a successful, inexpensive method with high specificity and sensitivity which can find wide applications for the rapid detection and identification of this virus.

### 3.2. Malacoherpesviridae

The herpesvirus known as ostreid herpesvirus-1 (OsHV-1) is frequently linked to huge mortalities in juvenile Pacific oysters (*Crassostrea gigas*). Several bivalve mollusc species have been linked to herpes-like viral infections worldwide. High mortality rates among juveniles and hatchery-reared larvae of several bivalve species have frequently been linked to such infections.

Traditionally, herpes-like virus infections’ diagnosis is performed by light microscopy to detect cytological abnormalities, followed by transmission electron microscopy, to visualise the virus. Due to molluscs’ inability to make antibodies, traditional serological techniques cannot be used to detect viruses in mollusc bivalves. Moreover, the absence of mollusc cell lines makes the viral isolation on cell cultures not possible. Due to these limitations, essential molecular diagnostic techniques such as PCR and other nucleic acid-based assays have been developed [126,127].

Both these methods possess advantages: the PCR is extremely sensitive and specific. On the other hand, in situ techniques are able to detect viral targets directly in the tissues, providing important information on the pathogen’s localisation at the cellular level.

Bivalve molluscs are filter-feeding animals, and throughout this activity, they can accumulate several pathogens, including viruses [128,129,130]. As a result, the association of viruses with bivalve molluscs can be due to both passive contamination and active infection, so to have suitable methods to distinguish between these different circumstances is crucial.

ISH techniques are able to stain specifically the OsHV-1 genomic sequence localising the virus in Pacific oyster tissues. Both digoxigenin (DIG)-labelled DNA and RNA probes have been applied to FFPE tissue sections. This method successfully localises the viral DNA in the gills, mantle, labial palps, digestive gland and gonads connective tissues. OsHV-1 was also detected within haemocytes in connective tissues surrounding the ova, within the ova and in myocytes in the heart and adductor muscle. However, this method cannot distinguish the reference and microvariant genotypes [131].

OsHV-1’s genome sequencing has made it possible to develop DNA-based diagnostic methods. In bivalve molluscs at different developmental stages, a PCR technique was employed to detect the OsHV-1 DNA. The PCR employed to detect OsHV-1 allowed to amplify DNA from an OsHV-1 variant [127]. Moreover, ostreid herpesvirus-1 microvariants were found in healthy *C. gigas*, and their potential role as reservoirs of infection was considered [132]. There are numerous assays, including conventional and qPCR tests with amplification targets in different regions of the OsHV-1 genome. Conventional PCR assays can target different regions of the genome and amplify both the reference and the OsHV-1 µVar genotype [127]. Quantitative PCR assays were developed using either SYBR Green or TaqMan probe chemistry targeting the same regions. The tests show high sensitivity and can find high throughput diagnostic applications, including surveillance, although exact diagnostic sensitivity and specificity have not yet been estimated [131].

A real-time PCR assay was used to investigate the presence of the OsHV-1 microvariant associated with mortality outbreaks in New Zealand Pacific oysters in 2010−2011, pointing out a discrepancy between OsHV-1 nucleic acid detection and the onset of mortality. Sections of Pacific oysters obtained from the same longitudinal investigation were also analysed by ISH. Interestingly, only a fair agreement was shown between ISH and real-time PCR results. Several reasons can explain this disparity: the type of material used for testing differed between the two tests, i.e., fresh vs. FFPE tissues, degradation of nucleic acid due to formalin fixation and the cross-linking of proteins around the remaining segments of nucleic acids may render them unavailable for detection. The ISH technique’s sensitivity could be also diminished by missing target sequences as well as inadequate DNA exposure during tissue pre-treatment processes such as proteinase K digestion. Finally, ISH only permits modest and linear signal amplification based on the number of DIG labels per strand and has restrictions imposed by antibody-enzyme binding and chromogen build-up, whereas PCR techniques are based on the exponential amplification of low copy numbers to a point where detection is possible. However, it should be underlined that real-time positive results, particularly those with high quantification cycle (Cq) values, must be interpreted cautiously when ascertaining true OsHV-1 infections in oysters. However, because of the high sensitivity of this molecular method, a real-time PCR may produce false-positive results due to the amplification of viral nucleic acids present in the water surrounding the oyster, outside the mantle and gills or within the gut lumen [133].

The development of quick, user-friendly and inexpensive detection methods is critical for the early diagnosis of OsHV-1 infection and ultimately reduce the impacts of the disease. Recent years have seen the emergence of isothermal DNA amplification techniques as a quick and effective tool for molecular diagnostics. These methods enable DNA amplification at one temperature, making it possible to combine them with miniaturised analytical devices to perform in situ tests. A simple, low-cost and miniaturised biosensor that combines recombinant polymerase amplification with an electrochemical read-out for the detection of OsHV-1 was constructed; this innovative biosensor configuration offers several advantages and makes this bioanalytical tool highly interesting for screening and quantification purposes [134].

To study the dynamics of viral pathogen transmission, their evolution and the emergence of new variants, whole-genome sequencing is frequently used providing crucial data to stakeholders for disease management. Unfortunately, because several aquatic viruses hardly replicate in vitro, their genomes are challenging to be characterised. So, due to the ongoing threat of disease emergence, it is essential to develop methodologies for the routine genome sequencing of aquatic viruses. This is especially true for pathogenic viruses that infect species of commercial interest that are frequently traded between production basins or countries [135].

With the above-described molecular procedures, some viruses, such as OsHV-1, can be difficult to be detected and identified when present in low amounts [127]. To enhance the recovery of OsHV-1 infectious particles from infected host tissues, a tangential flow filtration (TFF) technique was devised [135]. This method permitted a high molecular weight and high-quality viral DNA to be obtained for Illumina short-read and Nanopore long-read sequencing. Complete OsHV-1 genomes were assembled combining reads from both sequencing technologies. The developed TFF-based purification method, coupled with nanopore sequencing was revealed to be a promising approach to enabling in-field sequencing of unculturable aquatic DNA viruses.

Molecular techniques can also be adopted for molecular epidemiology. Mass mortality events associated with ostreid herpesvirus 1 have occurred in *C. gigas* in Australia. The genetic diversity of OsHV-1 in archival samples collected during mortality events in Australia was analysed, comparing the Australian viruses with international isolates previously published to investigate the source and transmission of the virus, finalised to improve the disease prevention and control procedures. OsHV-1 associated with Australian mortality outbreaks were distinct from the OsHV-1 reference genotype, µVar and other microvariants from other countries. The results show that OsHV-1 variants other than microvariants can also cause mass mortality events in *C. gigas*. Moreover, it was observed that a spatial clustering associated to different estuaries and a potential link between genotype/amino acid sequence and the prevalence and severity of mortality outbreaks that was different between these estuaries. This investigation suggests the presence of environmental reservoirs of OsHV-1 responsible for *C. gigas* mortality outbreaks in Australia [136].

## 4. *Nodaviridae*

The family *Nodaviridae* belongs to the order *Nodamuvirales* and it is currently divided into two genera: *Alphanodavirus* and *Betanodavirus*. Nodaviruses are small (29–32 nm in diameter), non-enveloped, icosahedral viruses whose genome is composed of two segments of positive strand single-stranded RNA named RNA1 and RNA2. The RNA1 encodes the viral RNA-dependent RNA polymerase (RdRp), whereas the RNA2 encodes the capsid protein. Alphanodaviruses primarily infect insects. They include *Nodamura virus* (NoV), *Flock house virus* (FHV), *Black beetle virus* (BBV), *Boolarra virus* (BoV) and *Pariacoto virus* (PaV) [137]. Betanodaviruses can infect over 50 fish species worldwide and they are the causative agents of viral nervous necrosis (VNN), also known as viral encephalopathy and retinopathy (VER). VNN outbreaks are characterised by clinical signs such as abnormal swimming such as erratic, frenzied or whirling swimming, loss of appetite, hyperinflation of the swim bladder and lightening or darkening of the coloration [138]. Histologically, the brain of the infected animals typically presents multiple intracytoplasmic vacuolations in the grey matter. Moreover, pyknosis, karyorrhexis, neuronal degeneration, inflammatory infiltration, congestion of the blood vessels and sometimes haemorrhages were also described in the nervous tissues of infected fish [139].

Before the 1990s, all known nodaviruses infected insects and were intensely studied because, due to their simple structure, they were considered a paradigm for understanding chemical biology [140]. However, the interest in developing efficient methods to study and detect these viruses further increased after the worldwide occurrence of several fish mortality outbreaks were associated with nodaviruses. Sequence analysis showed clearly that insect and fish viruses clustered separately according to significant differences in their genomes. In particular, a first study focused on the RNA2 segment showed the presence of a region conserved in all fish nodaviruses but absent in insect nodaviruses and the presence of a region more variable within the group of fish nodaviruses [141].

Betanodaviruses were, at first, identified by the name of the host species followed by “nervous necrosis virus” (NNV); however, the analysis of the viral genome showed that betanodaviruses infecting different species clustered together, forming a few genotypes. Phylogenetic analysis of the variable region within the RNA2 segment clusters betanodaviruses into four main genotypes [142]. Later research using the other viral genome fragment, the RNA1, confirmed the clustering of Betanodavirus into four main genotypes [143]. Currently, ICTV recognised the following official species: *Striped jack nervous necrosis virus* (SJNNV), *Tiger puffer nervous necrosis virus* (TPNNV), *Barfin flounder nervous necrosis virus* (BFNNV) and *Redspotted grouper nervous necrosis virus* (RGNNV) (https://ictv.global/taxonomy accessed on 2 February 2023).

More recently, further nodaviruses from invertebrate hosts other than insects have been detected and associated mainly with crustacean diseases and mortality, leading to a reconsideration of the taxonomy of the family *Nodaviridae* and the proposal of a new genus: *Gammanodavirus*. Indeed, the analysis of the genomes of these viruses shows they are clearly distinct from both alpha- and betanodaviruses. In particular, arthropod-infecting viruses have been included in this new proposed genus, such as Macrobrachium rosenbergii nodavirus (MrNV), Penaeus vannamei nodavirus (PvNV), Covert mortality nodavirus (CMNV) and Farfantepenaeus duorarum nodavirus (FdNV) [144,145,146].

### Betanodavirus

Betanodavirus was described for the first time from infected larval stripped jack (*Pseudocaranx dentex*). The name ‘striped jack nervous necrosis virus’ (SJNNV) was consequently adopted [147]. Subsequently, other agents of VNN were isolated from diseased fish species [148]. The present classification of the four species (SJNNV, RGNNV, TPNNV and BFNNV) was proposed for the first time by Nishizawa and colleagues in 1997 [142]. The first studies on host-specificity determinants in betanodaviruses discovered that SJNNV and TPNNV cause diseases in striped jack and tiger puffer (*Takifugu rubripes*), respectively; BFNNV infects cold-water species, including barfin flounder (*Verasper moseri*), turbot (*Scophthalmus maximus*) and Atlantic halibut (*Hippoglossus hippoglossus*). Conversely, RGNNV has a broad host range and has been isolated from a wide variety of warm-water fish species, especially groupers and sea bass. In order to determine the mechanisms underlying the host specificity, Iwamoto and colleagues [149] developed in vitro reassortants of the RGNNV and SJNNV species. Interestingly, they have found out that the host specificity of SJNNV and RGNNV is determined by the RNA2 segment coding the capsid protein. Moreover, host shifts of betanodaviruses have been attributed to RNA reassortments. In fact, Toffolo and colleagues in 2007 [143] discovered for the first time natural evidence of reassortant strains harbouring the RNA1-SJNNV and RNA2-RGNNV types.

Since betanodavirus classification has been accepted by the scientific community and the ICTV, several betanodavirus strains have been reported. Most of them cluster within one of the well-known genotypes, whereas a few diverge. Turbot nodavirus (TNV) was detected in turbot larvae showing classical signs of VER with abnormal swimming behaviour and high mortality levels. Despite the fact that the virus could not be isolated in cell culture and it resulted negative for an RT-PCR using primers designed to detect the Atlantic halibut nodavirus, a viral genome fragment could be amplified and sequenced with primers complementary to a more conserved region of RNA2. RNA2 analysis demonstrated that TNV differs from previously described fish nodaviruses and that it was not included in any of the four already known betanodavirus genotypes, so it was proposed as a fifth genotype within this genus [150]. On the other hand, betanodaviruses isolated from Atlantic cod (*Gadus morrhua*), haddock (*Melanogrammus aeglefinus*) and winter flounder (*Pseudopleuronectes americanus*) from eastern North America form a monophyletic group, named ‘Atlantic cod nervous necrosis virus’ (ACNNV), within the BFNNV genotype [151]. RNA2 analysis showed that all eastern North American betanodaviruses formed a distinct cluster from the European isolates. Furthermore, these betanodavirus isolates clustered more according to their geographical origin than their host species [151]. More recently, a new genotype has been proposed. A new type of betanodavirus was identified in shellfish and called Korean shellfish nervous necrosis virus (KSNNV) [152]. Phylogenetic analysis shows that KSNNVs form a distinct group only distantly related to other betanodavirus clusters, suggesting that KSNNV is an ancient member of the *Betanodavirus* genus [152].

The establishment of the striped snakehead cell line (SSN-1), derived from whole fry tissue of the striped snakehead (*Ophicephalus striatus*), allowed for the first effective isolation of a betanodavirus [153]. Even though NNV isolation in cell culture was a significant breakthrough and was considered the gold standard for detecting NNV infection for two decades, the procedure is time-consuming and labour-intensive, making it unsuitable for routine diagnosis, especially when processing large amounts of samples. For these reasons, molecular techniques have become increasingly relevant over the past few years. Indeed, several PCR-based techniques (RT-PCR, nested PCR and RT-qPCR), targeting RNA1, RNA2 or both genomic segments, have been reported [154]. Nowadays, the WOAH reference centre for viral encephalopathy and retinopathy suggests an RT-qPCR method for NNV diagnosis and surveillance [155] targeting the RNA1 genome segment, able to detect at least 50–100 copies of the target.

For a betanodavirus diagnosis, the isothermal amplification approach has also been explored. In particular, LAMP assays targeting the RNA2 segment of the RGNNV genotype have been developed in order to provide time- and cost-saving diagnostic tools. LAMP methods have the ability to amplify a target in one hour and do not require highly specialised laboratories. For these reasons, diagnostic methods using LAMP are also suitable for a company’s self-control procedures. However, the detection limit of the developed RT-LAMP applied for NNV detection was lower compared to qPCR assays [156,157,158].

Recently, evidence of natural reassortants between the RGNNV and SJNNV genotypes in southern Europe emerged from the sequencing of both genomic segments. In particular, both combinations of genomic segments, SJNNV/RGNNV and RGNNV/SJNNV (RNA1/RNA2), have been observed in viral isolates obtained from fish, although the second type has been detected more often [139,159,160,161]. The emergence of the reassortant strains and the large number of betanodavirus strains circulating at the same time in several geographical areas have promoted the application of molecular methods able to distinguish and identify the nature of the detected viruses. In this prospect, a multiplex RT-PCR assay able to distinguish among RGNNV, SJNNV and their reassortants has been developed, providing an easy, rapid and affordable molecular method to gain information about circulating NNV genotypes [5].

Moreover, the recent application of whole genome sequencing (WGS) to the study of aquatic viruses is gaining importance due to the large amount of information that it could generate. Accordingly, the WGS has permitted the identification of variable amino-acid residues probably correlated to the NNV virulence and pathogenicity thanks to the analysis of the NNV reassortant strains’ RdRp and capsid protein physico-chemical characteristics [162]. In particular, the amino acids (aa) 247 and 270 of capsid protein have been identified as putative viral receptor binding sites; accordingly, substitutions at these positions could affect the virus–host interaction. In addition, Biasini and colleagues [162] speculated on the potential existence of additional interesting residues that are all exposed on the outside of the structure of the trimeric P-domains. In particular, seven variable amino acids were identified and it was possible to speculate using protein-structure prediction that changes in these positions alter the hydrophobicity and the steric hindrance of the exposed surface region, changing the physiochemical properties of the capsid protein.

Molecular methods have also been successfully applied to identify specific areas of the central nervous system involved in NNV infection, providing a crucial understanding of the pathogenesis of this virus. Wang and colleagues [9], with a combined application of molecular-based methods such as real-time quantitative chain reaction (RT-qPCR) and FISH, were able to measure RGNNV content in different brain regions of an experimentally infected orange spotted grouper (*Epinephelus coioides*). The probe used in the FISH assay, targeting the capsid protein of RGNNV, revealed the presence of specific signals in the midbrain region. Furthermore, using quantitative (RT-qPCR) and qualitative (ISH) molecular methods, it is possible to detect the presence of the NNV genome as well as infectious particles in both nervous and non-nervous tissues and to study the viral uptake during the infection process. The application of ISH to an experimentally infected seven-band grouper (*Hyporthodus septemfasciatus*) was able to localise the RNA2 segment of the virus in the gill district during the first stages of NNV infection and in the blood–brain barrier, followed by the brain in the final stage of infection [163]. Lastly, the ISH has also been applied to investigate the NNV histopathology in other aquatic animal species, such as the big-belly seahorse (*Hippocampus abdominalis*). In particular, the application of different molecular methods (i.e., PCR detection, RT-qPCR and FISH) to the tissues of this aquatic vertebrate allowed the identification and localisation of a new NNV strain, closely related to the RGNNV genotype [164].

## 5. Discussion and Conclusions

With an average of 10^7^ virus-like particles per mL^−1^, an abundance typically ranging from 10^4^ to 10^8^ mL^–1^ and an order of magnitude greater than bacteria, viruses are widespread elements of aquatic ecosystems [3]. The majority of them are harmless to humans and animals and are essential for global and small-scale biogeochemical cycles, community structure, gene transfer and the evolution of aquatic organisms. Furthermore, some of them are even beneficial to animals and ecosystems infecting and then controlling aquatic pathogenic bacteria or harmful algae [3]. However, several aquatic viruses can infect animals, leading to diseases, especially when they are confined, such as in aquaculture facilities.

Traditional methods, such as viral isolation in cell cultures and antigen/antibody detection assays, have been widely applied to detect and study aquatic animals’ viruses, leading to the successful isolation, identification and understanding of several of them. These methods offer several advantages and are still indispensable many times. However, they also present several limitations, especially in their application to aquatic animals’ virology. As seen from the many reported examples, several fish viruses are not cultivable in cell cultures; in fact, some of them can only hardly or very slowly replicate in vitro; furthermore, the use of virus isolation in cell cultures is barred from the study of shellfish diseases due to the lack of suitable cell cultures. As a matter of fact, despite decades of attempts, not even a single cell line of marine molluscs or crustaceans is available [165,166]. Cell cultures are systems with a sensitive balance that are often subjected to toxic samples. Nevertheless, isolation in cell culture is often time-consuming, making it not ideal for routine processing, especially for surveillance when a number of samples need to be tested.

Serology is another traditional approach used to detect and study viral infections, with a limited application to aquatic animals’ viral diseases. Obviously, it is not accessible for animals that do not produce antibodies; however, it is often considered an inappropriate or unreliable method for viral diagnosis in finfish too [167,168]. In fact, fish antibody production following infection is slow, especially at low water temperatures, resulting in the absence of antibodies or a lack of homogeneity in antibody production in infected populations.

Molecular methods have significantly contributed to overcoming the limitations of traditional ones. Furthermore, intensive farming conditions and an increasing number of farmed fish species across the animal kingdom, including different phyla, have boosted the number of pathogens that humans and animal production have to deal with, requiring the quick development of new detection and study methods for novel unknown pathogens.

Molecular methods provide undoubtedly a powerful tool to detect, identify and characterise both known and unknown pathogens. A standard PCR protocol followed by the sequencing of purified amplicons is an effective method for both identifying well-known viruses and discovering new ones. PCR-based methods are taking place in surveillance programmes, either alongside or, in some cases, replacing the traditional methods. The PCR method is quick, allows the simultaneous examination of numerous samples, and is highly sensitive, with some PCR assays even claiming to be able to detect just a single copy of the target DNA.

On the other hand, sequencing techniques, especially next generation sequencing have recently become irreplaceable tools in emerging infectious disease research. Moreover, the increasing availability of sequences in databases makes it easier to compare and ascribe new sequences to specific taxa. A wide range of sequences is the starting point of all epidemiological studies and the identification of viruses at the species or even sub-species level can further refine epidemiological studies.

However, molecular methods also have limitations that are good to know. PCR assays are able to detect nucleic acids regardless of virus integrity; obviously the persistence of nucleic acids outside virions, especially RNA, is limited; however, their occasional findings, especially in new host species or uncommon substrates, needs to be ascertained. Furthermore, PCR positivity cannot be univocally associated with active viral replication or active infection and needs to be confirmed with the additional analysis. This constraint has to be kept in mind, especially with animals able to passively accumulate viruses in their flesh, such as bivalve molluscs. In this regard, in addition to traditional methods, which are not always applicable, other molecular methods can be helpful. Transcript detection, for example, can be pointed out through an RT-PCR for DNA viruses or by ISH using complementary RNA probes, demonstrating an active replication status.

Another critical point is the optimisation and validation of protocols. PCR assays are developed through the design of primers, which need to be as specific as possible but, at the same time, have to match a conserved sequence to avoid mismatches in the case of sequence variations. As a result, not all PCR protocols can provide adequate specificity and sensitivity fit for purpose, so especially for surveillance, it is fundamental to use validated protocols.

Despite these constraints, in the last few years the study of viral pathogens has relied on the invaluable contribution of molecular techniques primarily based on PCR, sequencing and in situ hybridisation gaining crucial knowledge about viral aquatic animal diseases. Finally, the study of viral aquatic animal diseases should not be separated from the development of new innovative techniques or the application of already available molecular methods in order to further increase their in-depth understanding.

## Data Availability

The data presented in this study are available from the corresponding author upon reasonable request.

## References

[1] Fonseca A., Laguardia-Nascimento M., Scotá Ferreira A., Pinto C., Pereira Freitas T., Rivetti Júnior A., Ferreira Homem V., Camargos M. (2022). Detection of Megalocytivirus in Oreochromis Niloticus and Pseudoplatystoma Corruscans in Brazil. Dis. Aquat. Organ..

[2] Shahzad S., Afzal M., Sikandar S., Afzal I., Jamal F. (2020). Polymerase Chain Reaction. Genetic Engineering—A Glimpse of Techniques and Applications.

[3] Jacquet S., Miki T., Noble R., Peduzzi P., Wilhelm S. (2010). Viruses in Aquatic Ecosystems: Important Advancements of the Last 20 Years and Prospects for the Future in the Field of Microbial Oceanography and Limnology. Adv. Oceanogr. Limnol..

[4] Pinheiro A.C.A.S., Volpe E., Principi D., Prosperi S., Ciulli S. (2016). Development of a Multiplex RT-PCR Assay for Simultaneous Detection of the Major Viruses That Affect Rainbow Trout (Oncorhynchus Mykiss). Aquac. Int..

[5] Errani F., Volpe E., Riera-Ferrer E., Caffara M., Padrós F., Gustinelli A., Fioravanti M., Ciulli S. (2022). Development and Diagnostic Validation of a One-Step Multiplex RT-PCR Assay as a Rapid Method to Detect and Identify Nervous Necrosis Virus (NNV) and Its Variants Circulating in the Mediterranean. PLoS ONE.

[6] Kubista M., Andrade J.M., Bengtsson M., Forootan A., Jonák J., Lind K., Sindelka R., Sjöback R., Sjögreen B., Strömbom L. (2006). The Real-Time Polymerase Chain Reaction. Mol. Asp. Med..

[7] Ciulli S., Pinheiro A.C.d.A.S.,  Volpe E., Moscato M., Jung T.S., Galeotti M., Stellino S., Farneti R., Prosperi S. (2015). Development and Application of a Real-Time PCR Assay for the Detection and Quantitation of Lymphocystis Disease Virus. J. Virol. Methods.

[8] Morley A.A. (2014). Digital PCR: A Brief History. Biomol. Detect. Quantif..

[9] Wang N., Zhang Z., Jing H., Zhang M., Wu S., Lin X. (2021). Development of a Novel Droplet Digital PCR Assay for the Sensitive Detection of Carp Edema Virus. Aquaculture.

[10] Zhao Y., Chen F., Li Q., Wang L., Fan C. (2015). Isothermal Amplification of Nucleic Acids. Chem. Rev..

[11] Yu Q., Liu M.-Z., Xiao H.-H., Yi Y., Cheng H., Putra D.-F., Li S.-Q., Li P.-F. (2020). Selection and Characterization of Aptamers for Specific Detection of Iridovirus Disease in Cultured Hybrid Grouper (Epinephelus Fuscoguttatus♀ × E. Lanceolatus♂). Chin. J. Anal. Chem..

[12] Pfankuche V.M., Hahn K., Bodewes R., Hansmann F., Habierski A., Haverkamp A.-K., Pfaender S., Walter S., Baechlein C., Postel A. (2018). Comparison of Different In Situ Hybridization Techniques for the Detection of Various RNA and DNA Viruses. Viruses.

[13] Cassidy A., Jones J. (2014). Developments in in Situ Hybridisation. Methods S. Diego Calif..

[14] Jansen G.J., Wiersma M., van Wamel W.J.B., Wijnberg I.D. (2021). Direct Detection of SARS-CoV-2 Antisense and Sense Genomic RNA in Human Saliva by Semi-Autonomous Fluorescence in Situ Hybridization: A Proxy for Contagiousness?. PLoS ONE.

[15] Arzul I., Nicolas J.-L., Davison A.J., Renault T. (2001). French Scallops: A New Host for Ostreid Herpesvirus-1. Virology.

[16] (2013). OIE CHAPTER 2.4.9 Infection with Ostreid Herpesvirus 1 Microvariant. Manual of Diagnostic Tests for Aquatic Animals.

[17] Snow M. (2011). The Contribution of Molecular Epidemiology to the Understanding and Control of Viral Diseases of Salmonid Aquaculture. Vet. Res..

[18] Tengs T., Rimstad E. (2017). Emerging Pathogens in the Fish Farming Industry and Sequencing-Based Pathogen Discovery. Dev. Comp. Immunol..

[19] Clouthier S., Anderson E., Kurath G., Breyta R. (2018). Molecular Systematics of Sturgeon Nucleocytoplasmic Large DNA Viruses. Mol. Phylogenet. Evol..

[20] Iyer L.M., Balaji S., Koonin E.V., Aravind L. (2006). Evolutionary Genomics of Nucleo-Cytoplasmic Large DNA Viruses. Virus Res..

[21] Chinchar V.G., Waltzek T.B., Subramaniam K. (2017). Ranaviruses and Other Members of the Family Iridoviridae: Their Place in the Virosphere. Virology.

[22] Wolf K., Gravell M., Malsberger R.G. (1966). Lymphocystis Virus: Isolation and Propagation in Centrarchid Fish Cell Lines. Science.

[23] Kurita J., Nakajima K. (2012). Megalocytiviruses. Viruses.

[24] Gray M., Miller D., Hoverman J. (2009). Ecology and Pathology of Amphibian Ranaviruses. Dis. Aquat. Organ..

[25] Jancovich J.K., Chinchar V.G., Hyatt A.D., Miyazaki T., Williams T., Zhang Q.Y. (2011). Family Iridoviridae. Virus Taxonomy, Ninth Report of the International Committee on Taxonomy of Viruses.

[26] Volpatti D., Ciulli S. (2022). Chapter 15 Lymphocystis Virus Disease. Aquaculture Pathophysiology Volume I. Fish Diseases.

[27] Doszpoly A., Kaján G.L., Puentes R., Perretta A. (2020). Complete Genome Sequence and Analysis of a Novel Lymphocystivirus Detected in Whitemouth Croaker (Micropogonias Furnieri): Lymphocystis Disease Virus 4. Arch. Virol..

[28] Benkaroun J., Bergmann S.M., Römer-Oberdörfer A., Demircan M.D., Tamer C., Kachh G.R., Weidmann M. (2022). New Insights into Lymphocystis Disease Virus Genome Diversity. Viruses.

[29] Mönttinen H.A.M., Bicep C., Williams T.A., Hirt R.P. (2021). The Genomes of Nucleocytoplasmic Large DNA Viruses: Viral Evolution Writ Large. Microb. Genom..

[30] Kitamura S.-I., Jung S.-J., Kim W.-S., Nishizawa T., Yoshimizu M., Oh M.-J. (2006). A New Genotype of Lymphocystivirus, LCDV-RF, from Lymphocystis Diseased Rockfish. Arch. Virol..

[31] Cano I., Valverde E.J., Lopez-Jimena B., Alonso M.C., Garcia-Rosado E., Sarasquete C., Borrego J.J., Castro D. (2010). A New Genotype of Lymphocystivirus Isolated from Cultured Gilthead Seabream, Sparus Aurata L., and Senegalese Sole, Solea Senegalensis (Kaup). J. Fish Dis..

[32] Shawky M., Taha E., Ahmed B., Mahmoud M.A., Abdelaziz M., Faisal M., Yousif A. (2021). Initial Evidence That Gilthead Seabream (*Sparus Aurata* L.) Is a Host for Lymphocystis Disease Virus Genotype I. Animals.

[33] Kvitt H., Heinisch G., Diamant A. (2008). Detection and Phylogeny of Lymphocystivirus in Sea Bream Sparus Aurata Based on the DNA Polymerase Gene and Major Capsid Protein Sequences. Aquaculture.

[34] Wu R.-H., Tang X.-Q., Sheng X.-Z., Zhan W.-B. (2015). Tissue Distribution of the 27.8 KDa Receptor and Its Dynamic Expression in Response to Lymphocystis Disease Virus Infection in Flounder (Paralichthys Olivaceus). J. Comp. Pathol..

[35] Volpe E., Farneti R., Moscato M., Prosperi S., Ciulli S. (2015). Distribuzione Di Lymphocystivirus in Organi Target e Non Target Di Orate (Sparus Aurata) Naturalmente Infette. Ittiopatologia.

[36] Cano I., Ferro P., Alonso M.C., Sarasquete C., Garcia-Rosado E., Borrego J.J., Castro D. (2009). Application of in Situ Detection Techniques to Determine the Systemic Condition of Lymphocystis Disease Virus Infection in Cultured Gilt-Head Seabream, *Sparus Aurata* L. J. Fish Dis..

[37] Valverde E.J., Cano I., Labella A., Borrego J.J., Castro D. (2016). Application of a New Real-Time Polymerase Chain Reaction Assay for Surveillance Studies of Lymphocystis Disease Virus in Farmed Gilthead Seabream. BMC Vet. Res..

[38] Li Q., Yue Z., Liu H., Liang C., Zheng X., Zhao Y., Chen X., Xiao X., Chen C. (2010). Development and Evaluation of a Loop-Mediated Isothermal Amplification Assay for Rapid Detection of Lymphocystis Disease Virus. J. Virol. Methods.

[39] Valverde E.J., Borrego J.J., Sarasquete M.C., Ortiz-Delgado J.B., Castro D. (2017). Target Organs for Lymphocystis Disease Virus Replication in Gilthead Seabream (Sparus Aurata). Vet. Res..

[40] Go J., Waltzek T., Subramaniam K., Yun S., Groff J., Anderson I., Chong R., Shirley I., Schuh J., Handlinger J. (2016). Detection of Infectious Spleen and Kidney Necrosis Virus (ISKNV) and Turbot Reddish Body Iridovirus (TRBIV) from Archival Ornamental Fish Samples. Dis. Aquat. Organ..

[41] Waltzek T., Marty G., Alfaro M., Bennett W., Garver K., Haulena M., Weber ES I., Hedrick R. (2012). Systemic Iridovirus from Threespine Stickleback Gasterosteus Aculeatus Represents a New Megalocytivirus Species (Family Iridoviridae). Dis. Aquat. Organ..

[42] de Groof A., Guelen L., Deijs M., van der Wal Y., Miyata M., Ng K.S., van Grinsven L., Simmelink B., Biermann Y., Grisez L. (2015). A Novel Virus Causes Scale Drop Disease in Lates Calcarifer. PLoS Pathog..

[43] Halaly M.A., Subramaniam K., Koda S.A., Popov V.L., Stone D., Way K., Waltzek T.B. (2019). Characterization of a Novel Megalocytivirus Isolated from European Chub (Squalius Cephalus). Viruses.

[44] Canuti M., Eis-Huebinger A.M., Deijs M., de Vries M., Drexler J.F., Oppong S.K., Müller M.A., Klose S.M., Wellinghausen N., Cottontail V.M. (2011). Two Novel Parvoviruses in Frugivorous New and Old World Bats. PLoS ONE.

[45] Tan L.V., Van Doorn H.R., Van der Hoek L., Minh Hien V., Jebbink M.F., Quang Ha D., Farrar J., Van Vinh Chau N., de Jong M.D. (2011). Random PCR and Ultracentrifugation Increases Sensitivity and Throughput of VIDISCA for Screening of Pathogens in Clinical Specimens. J. Infect. Dev. Ctries..

[46] van der Hoek L., Pyrc K., Jebbink M.F., Vermeulen-Oost W., Berkhout R.J.M., Wolthers K.C., Wertheim-van Dillen P.M.E., Kaandorp J., Spaargaren J., Berkhout B. (2004). Identification of a New Human Coronavirus. Nat. Med..

[47] Inouye K., Yamano K., Maeno Y., Nakajima K., Matsuoka M., Wada Y., Sorimachi M. (1992). Iridovirus Infection of Cultured Red Sea Bream, Pagrus major. Fish Pathol..

[48] Stephens F.J., Jones J.B., Hillier P. (2009). Ornamental Fish Testing Project: Final Report.

[49] (2021). OIE CHAPTER 2.3.7 Red Sea Bream Iridoviral Disease. Manual of Diagnostic Tests for Aquatic Animals.

[50] Kurita J., Nakajima K., Hirono I., Aoki T. (1998). Polymerase Chain Reaction (PCR) Amplification of DNA of Red Sea Bream [Pagrus Major] Iridovirus (RSIV). Fish Pathol. Jpn..

[51] Rimmer A.E., Becker J.A., Tweedie A., Whittington R.J. (2012). Development of a Quantitative Polymerase Chain Reaction (QPCR) Assay for the Detection of Dwarf Gourami Iridovirus (DGIV) and Other Megalocytiviruses and Comparison with the Office International Des Epizooties (OIE) Reference PCR Protocol. Aquaculture.

[52] Caipang C.M.A., Haraguchi I., Ohira T., Hirono I., Aoki T. (2004). Rapid Detection of a Fish Iridovirus Using Loop-Mediated Isothermal Amplification (LAMP). J. Virol. Methods.

[53] Zhang Q., Shi C., Huang J., Jia K., Chen X., Liu H. (2009). Rapid Diagnosis of Turbot Reddish Body Iridovirus in Turbot Using the Loop-Mediated Isothermal Amplification Method. J. Virol. Methods.

[54] Ding W.C., Chen J., Shi Y.H., Lu X.J., Li M.Y. (2010). Rapid and Sensitive Detection of Infectious Spleen and Kidney Necrosis Virus by Loop-Mediated Isothermal Amplification Combined with a Lateral Flow Dipstick. Arch. Virol..

[55] Sukonta T., Senapin S., Meemetta W., Chaijarasphong T. (2022). CRISPR-based Platform for Rapid, Sensitive and Field-deployable Detection of Scale Drop Disease Virus in Asian Sea Bass (*Lates Calcarifer*). J. Fish Dis..

[56] Sudthongkong C., Miyata M., Miyazaki T. (2002). Viral DNA Sequences of Genes Encoding the ATPase and the Major Capsid Protein of Tropical Iridovirus Isolates Which Are Pathogenic to Fishes in Japan, South China Sea and Southeast Asian Countries. Arch. Virol..

[57] Adkinson M.A., Cambre M., Hedrick R.P. (1998). Identification of an Iridovirus in Russian Sturgeon (*Acipenser Guldenstadi*) from Northern Europe. Bull. Eur. Assoc. Fish Pathol. UK.

[58] Drennan J.D., Lapatra S.E., Samson C.A., Ireland S., Eversman K.F., Cain K.D. (2007). Evaluation of Lethal and Non-Lethal Sampling Methods for the Detection of White Sturgeon Iridovirus Infection in White Sturgeon, Acipenser Transmontanus (Richardson). J. Fish Dis..

[59] Ciulli S., Volpe E., Sirri R., Passalacqua P.L., Cesa Bianchi F., Serratore P., Mandrioli L. (2016). Outbreak of Mortality in Russian (Acipenser Gueldenstaedtii) and Siberian (Acipenser Baerii) Sturgeons Associated with Sturgeon Nucleo-Cytoplasmatic Large DNA Virus. Vet. Microbiol..

[60] Clouthier S., VanWalleghem E., Anderson E. (2015). Sturgeon Nucleo-Cytoplasmic Large DNA Virus Phylogeny and PCR Tests. Dis. Aquat. Organ..

[61] Bigarré L., Lesne M., Lautraite A., Chesneau V., Leroux A., Jamin M., Boitard P.M., Toffan A., Prearo M., Labrut S. (2017). Molecular Identification of Iridoviruses Infecting Various Sturgeon Species in Europe. J. Fish Dis..

[62] Raoult D., Audic S., Robert C., Abergel C., Renesto P., Ogata H., La Scola B., Suzan M., Claverie J.-M. (2004). The 1.2-Megabase Genome Sequence of Mimivirus. Science.

[63] Abrahão J., Silva L., Silva L.S., Khalil J.Y.B., Rodrigues R., Arantes T., Assis F., Boratto P., Andrade M., Kroon E.G. (2018). Tailed Giant Tupanvirus Possesses the Most Complete Translational Apparatus of the Known Virosphere. Nat. Commun..

[64] Claverie J.-M., Abergel C. (2018). Mimiviridae: An Expanding Family of Highly Diverse Large DsDNA Viruses Infecting a Wide Phylogenetic Range of Aquatic Eukaryotes. Viruses.

[65] Boutier M., Gao Y., Donohoe O., Vanderplasschen A. (2019). Current Knowledge and Future Prospects of Vaccines against Cyprinid Herpesvirus 3 (CyHV-3). Fish Shellfish. Immunol..

[66] Hanson L., Dishon A., Kotler M. (2011). Herpesviruses That Infect Fish. Viruses.

[67] Poli G., Dall’Ara P., Martino P.A., Rosati S. (2017). Microbiologia e Immunologia Veterinaria.

[68] Umene K., Sakaoka H. (1999). Evolution of Herpes Simplex Virus Type 1 under Herpesviral Evolutionary Processes. Arch. Virol..

[69] Sierra E., Fernández A., Fernández-Maldonado C., Sacchini S., Felipe-Jiménez I., Segura-Göthlin S., Colom-Rivero A., Câmara N., Puig-Lozano R., Rambaldi A.M. (2022). Molecular Characterization of Herpesviral Encephalitis in Cetaceans: Correlation with Histopathological and Immunohistochemical Findings. Animals.

[70] Lovy J., Friend S.E. (2014). Cyprinid Herpesvirus-2 Causing Mass Mortality in Goldfish: Applying Electron Microscopy to Histological Samples for Diagnostic Virology. Dis. Aquat. Organ..

[71] Doszpoly A., Benkő M., Bovo G., LaPatra S.E., Harrach B. (2011). Comparative Analysis of a Conserved Gene Block from the Genome of the Members of the Genus *Ictalurivirus*. Intervirology.

[72] Freitas J.T., Subramaniam K., Kelley K.L., Marcquenski S., Groff J., Waltzek T.B. (2016). Genetic Characterization of Esocid Herpesvirus 1 (EsHV1). Dis. Aquat. Organ..

[73] Sunarto A., McColl K.A., Crane M.S.J., Sumiati T., Hyatt A.D., Barnes A.C., Walker P.J. (2011). Isolation and Characterization of Koi Herpesvirus (KHV) from Indonesia: Identification of a New Genetic Lineage: Indonesian KHV Characterization. J. Fish Dis..

[74] Waltzek T.B., Kelley G.O., Alfaro M.E., Kurobe T., Davison A.J., Hedrick R.P. (2009). Phylogenetic Relationships in the Family Alloherpesviridae. Dis. Aquat. Organ..

[75] Pellett P.E., Davison A.J., Ederle R., Ehlers B., Hayward G.S., Lacoste V., Minson A.C., Nicholas J., Roizman B., Studdert M.J. (2011). Order Herpesvirales. Virus Taxonomy, Ninth Report of the International Committee on Taxonomy of Viruses.

[76] Gotesman M., Kattlun J., Bergmann S.M., El-Matbouli M. (2013). CyHV-3: The Third Cyprinid Herpesvirus. Dis. Aquat. Organ..

[77] McAllister P.E., Lidgerding B.C., Herman R.L., Hoyer L.C., Hankins J. (1985). Viral Diseases of Fish: First Report of Carp Pox in Golden Ide (Leuciscus Idus) in North America. J. Wildl. Dis..

[78] Hedrick R.P., Groff J.M., Okihiro M.S., McDowell T.S. (1990). Herpesviruses Detected in Papillomatous Skin Growths of Koi Carp (Cyprinus Carpio). J. Wildl. Dis..

[79] Sano N., Sano M., Sano T., Hondo R. (1992). Herpesvirus Cyprini: Detection of the Viral Genome by in Situ Hybridization. J. Fish Dis..

[80] Sano N., Moriwake M., Hondo R., Sano T. (1993). Herpesvirus Cyprini: A Search for Viral Genome in Infected Fish by Infected Fish by in Situ Hybridization. J. Fish Dis..

[81] Way K., Dixon P.F. (2017). Koi Herpesvirus Disease. Fish Viruses and Bacteria: Pathobiology and Protection.

[82] Lievens B., Frans I., Heusdens C., Justé A., Jonstrup S.P., Lieffrig F., Willems K.A. (2011). Rapid Detection and Identification of Viral and Bacterial Fish Pathogens Using a DNA Array-Based Multiplex Assay. J. Fish Dis..

[83] Sirri R., Ciulli S., Barbé T., Volpe E., Lazzari M., Franceschini V., Errani F., Sarli G., Mandrioli L. (2018). Detection of Cyprinid Herpesvirus 1 DNA in Cutaneous Squamous Cell Carcinoma of Koi Carp (Cyprinus Carpio). Vet. Dermatol..

[84] Yuasa K., Kurita J., Kawana M., Kiryu I., Oseko N., Sano M. (2012). Development of MRNA-Specific RT-PCR for the Detection of Koi Herpesvirus (KHV) Replication Stage. Dis. Aquat. Organ..

[85] Xu J., Zeng L., Zhang H., Zhou Y., Ma J., Fan Y. (2013). Cyprinid Herpesvirus 2 Infection Emerged in Cultured Gibel Carp, Carassius Auratus Gibelio in China. Vet. Microbiol..

[86] Jiang N., Xu J., Ma J., Fan Y., Zhou Y., Liu W., Zeng L. (2015). Histopathology and Ultrastructural Pathology of Cyprinid Herpesvirus II (CyHV-2) Infection in Gibel Carp, Carassius Auratus Gibelio. Wuhan Univ. J. Nat. Sci..

[87] Fichi G., Susini F., Cocumelli C., Cersini A., Salvadori M., Guarducci M., Cardeti G. (2016). New Detection of Cyprinid Herpesvirus 2 in Mass Mortality Event of Carassius Carassius (L.), in Italy. J. Fish Dis..

[88] Tang R., Lu L., Wang B., Yu J., Wang H. (2020). Identification of the Immediate-Early Genes of Cyprinid Herpesvirus 2. Viruses.

[89] Wang H., Xu L., Lu L. (2016). Detection of Cyprinid Herpesvirus 2 in Peripheral Blood Cells of Silver Crucian Carp, Carassius Auratus Gibelio (Bloch), Suggests Its Potential in Viral Diagnosis. J. Fish Dis..

[90] Ding Z., Xia S., Zhao Z., Xia A., Shen M., Tang J., Xue H., Geng X., Yuan S. (2014). Histopathological Characterization and Fluorescence in Situ Hybridization of Cyprinid Herpesvirus 2 in Cultured Prussian Carp, Carassius Auratus Gibelio in China. J. Virol. Methods.

[91] Ma J., Jiang N., LaPatra S.E., Jin L., Xu J., Fan Y., Zhou Y., Zeng L. (2015). Establishment of a Novel and Highly Permissive Cell Line for the Efficient Replication of Cyprinid Herpesvirus 2 (CyHV-2). Vet. Microbiol..

[92] Ito T., Kurita J., Ozaki A., Sano M., Fukuda H., Ototake M. (2013). Growth of Cyprinid Herpesvirus 2 (CyHV-2) in Cell Culture and Experimental Infection of Goldfish Carassius Auratus. Dis. Aquat. Organ..

[93] Lu J., Xu D., Lu L. (2018). A Novel Cell Line Established from Caudal Fin Tissue of Carassius Auratus Gibelio Is Susceptible to Cyprinid Herpesvirus 2 Infection with the Induction of Apoptosis. Virus Res..

[94] Engelsma M.Y., Way K., Dodge M.J., Voorbergen-Laarman M., Panzarin V., Abbadi M., El-Matbouli M., Frank Skall H., Kahns S., Stone D.M. (2013). Detection of Novel Strains of Cyprinid Herpesvirus Closely Related to Koi Herpesvirus. Dis. Aquat. Organ..

[95] Goodwin A., Merry G., Sadler J. (2006). Detection of the Herpesviral Hematopoietic Necrosis Disease Agent (Cyprinid Herpesvirus 2) in Moribund and Healthy Goldfish: Validation of a Quantitative PCR Diagnostic Method. Dis. Aquat. Organ..

[96] He J., Shi X., Yu L., Zheng X., Lan W., Jia P., Wang J., Liu H. (2013). Development and Evaluation of a Loop-Mediated Isothermal Amplification Assay for Diagnosis of Cyprinid Herpesvirus 2. J. Virol. Methods.

[97] Preena P.G., Kumar T.V.A., Johny T.K., Dharmaratnam A., Swaminathan T.R. (2022). Quick Hassle-Free Detection of Cyprinid Herpesvirus 2 (CyHV-2) in Goldfish Using Recombinase Polymerase Amplification-Lateral Flow Dipstick (RPA-LFD) Assay. Aquac. Int..

[98] Wang H., Sun M., Xu D., Podok P., Xie J., Jiang Y., Lu L. (2018). Rapid Visual Detection of Cyprinid Herpesvirus 2 by Recombinase Polymerase Amplification Combined with a Lateral Flow Dipstick. J. Fish Dis..

[99] Yang J., Wen J., Xiao S., Wei C., Yu F., Roengjit P., Lu L., Wang H. (2022). Complete Genome and Molecular Characterization of a New Cyprinid Herpesvirus 2 (CyHV-2) SH-01 Strain Isolated from Cultured Crucian Carp. Viruses.

[100] Chong R. (2022). Chapter 14 Koi Herpesvirus Disease. Aquaculture Pathophysiology Volume I. Fish Diseases.

[101] Hanson L., Doszpoly A., Van Beurden S.J., de Oliveira Viadanna P.H., Waltzek T.B. (2016). Chapter 9 Alloherpesviruses of Fish. Aquaculture Virology.

[102] Bercovier H., Fishman Y., Nahary R., Sinai S., Zlotkin A., Eyngor M., Gilad O., Eldar A., Hedrick R.P. (2005). Cloning of the Koi Herpesvirus (KHV) Gene Encoding Thymidine Kinase and Its Use for a Highly Sensitive PCR Based Diagnosis. BMC Microbiol..

[103] Gray W.L., Mullis L., LaPatra S.E., Groff J.M., Goodwin A. (2002). Detection of Koi Herpesvirus DNA in Tissues of Infected Fish. J. Fish Dis..

[104] Yuasa K., Sano M., Kurita J., Ito T., Iida T. (2005). Improvement of a PCR Method with the Sph I-5 Primer Set for the Detection of Koi Herpesvirus (KHV). Fish Pathol..

[105] Yuasa K., Sano M. (2009). Koi Herpesvirus: Status of Outbreaks, Diagnosis, Surveillance, and Research. Isr. J. Aquac. Bamidgeh.

[106] (2022). OIE CHAPTER 2.3.6 Infection with Koi Herpesvirus. Manual of Diagnostic Tests for Aquatic Animals.

[107] Yuasa K., Kawana M., Ito T., Kiryu I., Oseko N., Sano M. (2022). Intra Vitam Assays for Detecting Fish Infected with Cyprinid Herpesvirus 3 (CyHV-3). Dis. Aquat. Organ..

[108] Soliman H., El-Matbouli M. (2018). Rapid Detection and Differentiation of Carp Oedema Virus and Cyprinid Herpes Virus-3 in Koi and Common Carp. J. Fish Dis..

[109] Boutier M., Ronsmans M., Rakus K., Jazowiecka-Rakus J., Vancsok C., Morvan L., Peñaranda M.M.D., Stone D.M., Way K., van Beurden S.J. (2015). Cyprinid Herpesvirus 3: An Archetype of Fish Alloherpesviruses. Adv. Virus Res..

[110] Cano I., Worswick J., Mulhearn B., Stone D., Wood G., Savage J., Paley R. (2021). A Seasonal Study of Koi Herpesvirus and Koi Sleepy Disease Outbreaks in the United Kingdom in 2018 Using a Pond-Side Test. Animals.

[111] Thorstensen M.J., Vandervelde C.A., Bugg W.S., Michaleski S., Vo L., Mackey T.E., Lawrence M.J., Jeffries K.M. (2022). Non-Lethal Sampling Supports Integrative Movement Research in Freshwater Fish. Front. Genet..

[112] Monaghan S.J., Thompson K.D., Adams A., Kempter J., Bergmann S.M. (2015). Examination of the Early Infection Stages of Koi Herpesvirus (KHV) in Experimentally Infected Carp, Cyprinus Carpio L. Using in Situ Hybridization. J. Fish Dis..

[113] Doszpoly A., Kovács E.R., Bovo G., LaPatra S.E., Harrach B., Benkő M. (2008). Molecular Confirmation of a New Herpesvirus from Catfish (Ameiurus Melas) by Testing the Performance of a Novel PCR Method, Designed to Target the DNA Polymerase Gene of Alloherpesviruses. Arch. Virol..

[114] Doszpoly A., Shchelkunov I. (2010). Partial Genome Analysis of Siberian Sturgeon Alloherpesvirus Suggests Its Close Relation to AciHV-2—Short Communication. Acta Vet. Hung..

[115] Radosavljević V., Milićević V., Maksimović-Zorić J., Veljović L., Nešić K., Pavlović M., Pelić D.L., Marković Z. (2019). Sturgeon Diseases in Aquaculture. Arch. Vet. Med..

[116] Doszpoly A., Kalabekov I.M., Breyta R., Shchelkunov I.S. (2017). Isolation and Characterization of an Atypical Siberian Sturgeon Herpesvirus Strain in Russia: Novel North American *Acipenserid Herpesvirus 2* Strain in Europe?. J. Fish Dis..

[117] Kelley G.O., Waltzek T.B., McDowell T.S., Yun S.C., LaPatra S.E., Hedrick R.P. (2005). Genetic Relationships among Herpes-Like Viruses Isolated from Sturgeon. J. Aquat. Anim. Health.

[118] Kurobe T., Kelley G.O., Waltzek T.B., Hedrick R.P. (2008). Revised Phylogenetic Relationships among Herpesviruses Isolated from Sturgeons. J. Aquat. Anim. Health.

[119] Ciulli S., Volpe E., Sirri R., Tura G., Errani F., Zamperin G., Toffan A., Silvi M., Renzi A., Abbadi M. (2020). Multifactorial Causes of Chronic Mortality in Juvenile Sturgeon (Huso Huso). Animals.

[120] Hedrick R., McDowell T., Gilad O., Adkison M., Bovo G. (2003). Systemic Herpes-like Virus in Catfish *Ictalurus Melas* (Italy) Differs from Ictalurid Herpesvirus 1 (North America). Dis. Aquat. Organ..

[121] Alborali L., Paternello P., Lavazza A., Caggiano M., Lombardi G. (1996). Descrizione Di Un Episodio Di Linfocisti Nel Sarago (Diplodus Puntazzo). Boll. Soc. Ital. Patol. Ittica.

[122] Thompson D.J., Khoo L.H., Wise D.J., Hanson L.A. (2005). Evaluation of Channel Catfish Virus Latency on Fingerling Production Farms in Mississippi. J. Aquat. Anim. Health.

[123] Gray W.L., Williams R.J., Jordan R.L., Griffin B.R. (1999). Detection of Channel Catfish Virus DNA in Latently Infected Catfish. J. Gen. Virol..

[124] Goodwin A.E., Marecaux E. (2010). Validation of a QPCR Assay for the Detection of Ictalurid Herpesvirus-2 (IcHV-2) in Fish Tissues and Cell Culture Supernatants. J. Fish Dis..

[125] Hao K., Liu H.-Y., Chen X.-H., Yuan J.-F., Li L.-J., Bian W.-J., Zhao Z. (2021). Real-Time fluorescent loop mediated isothermal amplification for detection of channel catfish virus. Acta Hydrobiol. Sin..

[126] Barbosa-Solomieu V., Miossec L., Vázquez-Juárez R., Ascencio-Valle F., Renault T. (2004). Diagnosis of Ostreid Herpesvirus 1 in Fixed Paraffin-Embedded Archival Samples Using PCR and in Situ Hybridisation. J. Virol. Methods.

[127] Batista F.M., Arzul I., Pepin J.-F., Ruano F., Friedman C.S., Boudry P., Renault T. (2007). Detection of Ostreid Herpesvirus 1 DNA by PCR in Bivalve Molluscs: A Critical Review. J. Virol. Methods.

[128] Carter M.J. (2005). Enterically Infecting Viruses: Pathogenicity, Transmission and Significance for Food and Waterborne Infection. J. Appl. Microbiol..

[129] Errani F., Ponti M., Volpe E., Ciulli S. (2021). Spatial and Seasonal Variability of Human and Fish Viruses in Mussels inside and Offshore of Ravenna’s Harbour (Adriatic Sea, Italy). J. Appl. Microbiol..

[130] Volpe E., Pagnini N., Serratore P., Ciulli S. (2017). Fate of Redspotted Grouper Nervous Necrosis Virus (RGNNV) in Experimentally Challenged Manila Clam Ruditapes Philippinarum. Dis. Aquat. Organ..

[131] Furhmann M., Patirana E., de Kantzou M., Hick P. (2022). Chapter 63 Ostreid Herpesvirus Disease. Aquaculture Pathophysiology Volume II. Crustacean and Molluscan Diseases.

[132] Evans O., Paul-Pont I., Whittington R.J. (2017). Detection of Ostreid Herpesvirus 1 Microvariant DNA in Aquatic Invertebrate Species, Sediment and Other Samples Collected from the Georges River Estuary, New South Wales, Australia. Dis. Aquat. Organ..

[133] Bueno R., Perrott M., Dunowska M., Brosnahan C., Johnston C. (2016). In Situ Hybridization and Histopathological Observations during Ostreid Herpesvirus-1-Associated Mortalities in Pacific Oysters Crassostrea Gigas. Dis. Aquat. Organ..

[134] Toldrà A., Furones M.D., O’Sullivan C.K., Campàs M. (2020). Detection of Isothermally Amplified Ostreid Herpesvirus 1 DNA in Pacific Oyster (Crassostrea Gigas) Using a Miniaturised Electrochemical Biosensor. Talanta.

[135] Dotto-Maurel A., Pelletier C., Morga B., Jacquot M., Faury N., Dégremont L., Bereszczynki M., Delmotte J., Escoubas J.-M., Chevignon G. (2022). Evaluation of Tangential Flow Filtration Coupled to Long-Read Sequencing for Ostreid Herpesvirus Type 1 Genome Assembly. Microb. Genom..

[136] Trancart S., Tweedie A., Liu O., Paul-Pont I., Hick P., Houssin M., Whittington R.J. (2023). Diversity and Molecular Epidemiology of Ostreid Herpesvirus 1 in Farmed Crassostrea Gigas in Australia: Geographic Clusters and Implications for “Microvariants” in Global Mortality Events. Virus Res..

[137] Thiéry R., Johnson K.L., Nakai T., Schneemann A., Bonami J.R., Lightner D.V. (2011). Family Nodaviridae. Virus Taxonomy, Ninth Report of the International Committee on Taxonomy of Viruses.

[138] Bandín I., Souto S. (2020). Betanodavirus and VER Disease: A 30-Year Research Review. Pathogens.

[139] Volpe E., Gustinelli A., Caffara M., Errani F., Quaglio F., Fioravanti M.L., Ciulli S. (2020). Viral Nervous Necrosis Outbreaks Caused by the RGNNV/SJNNV Reassortant Betanodavirus in Gilthead Sea Bream (Sparus Aurata) and European Sea Bass (Dicentrarchus Labrax). Aquaculture.

[140] Schneemann A., Reddy V., Johnson J.E. (1998). The Structure and Function of Nodavirus Particles: A Paradigm for Understanding Chemical Biology. Advances in Virus Research.

[141] Nishizawa T., Mori K.-I., Furuhashi M., Nakai T., Furusawa I., Muroga K. (1995). Comparison of the Coat Protein Genes of Five Fish Nodaviruses, the Causative Agents of Viral Nervous Necrosis in Marine Fish. J. Gen. Virol..

[142] Nishizawa T., Furuhashi M., Nagai T., Nakai T., Muroga K. (1997). Genomic Classification of Fish Nodaviruses by Molecular Phylogenetic Analysis of the Coat Protein Gene. Appl. Environ. Microbiol..

[143] Toffolo V., Negrisolo E., Maltese C., Bovo G., Belvedere P., Colombo L., Valle L.D. (2007). Phylogeny of Betanodaviruses and Molecular Evolution of Their RNA Polymerase and Coat Proteins. Mol. Phylogenet. Evol..

[144] Ho K.L., Gabrielsen M., Beh P.L., Kueh C.L., Thong Q.X., Streetley J., Tan W.S., Bhella D. (2018). Structure of the Macrobrachium Rosenbergii Nodavirus: A New Genus within the Nodaviridae?. PLoS Biol..

[145] NaveenKumar S., Shekar M., Karunasagar I., Karunasagar I. (2013). Genetic Analysis of RNA1 and RNA2 of Macrobrachium Rosenbergii Nodavirus (MrNV) Isolated from India. Virus Res..

[146] Johnson K.L., Moore J.S. (2021). Nodaviruses of Invertebrates and Fish (Nodaviridae). Encyclopedia of Virology.

[147] Mori K., Nakai T., Muroga K., Arimoto M., Mushiake K., Furusawa I. (1992). Properties of a New Virus Belonging to Nodaviridae Found in Larval Striped Jack (Pseudocaranx Dentex) with Nervous Necrosis. Virology.

[148] Munday B.L., Kwang J., Moody N. (2002). Betanodavirus Infections of Teleost Fish: A Review. J. Fish Dis..

[149] Iwamoto T., Okinaka Y., Mise K., Mori K.-I., Arimoto M., Okuno T., Nakai T. (2004). Identification of Host-Specificity Determinants in Betanodaviruses by Using Reassortants between Striped Jack Nervous Necrosis Virus and Sevenband Grouper Nervous Necrosis Virus. J. Virol..

[150] Johansen R., Sommerset I., Tørud B., Korsnes K., Hjortaas M.J., Nilsen F., Nerland A.H., Dannevig B.H. (2004). Characterization of Nodavirus and Viral Encephalopathy and Retinopathy in Farmed Turbot, Scophthalmus Maximus (L.). J. Fish Dis..

[151] Gagné N., Johnson S.C., Cook-Versloot M., MacKinnon A.M., Olivier G. (2004). Molecular Detection and Characterization of Nodavirus in Several Marine Fish Species from the Northeastern Atlantic. Dis. Aquat. Organ..

[152] Kim Y.C., Kwon W.J., Min J.G., Kim K.I., Jeong H.D. (2019). Complete Genome Sequence and Pathogenic Analysis of a New Betanodavirus Isolated from Shellfish. J. Fish Dis..

[153] Frerichs G.N., Rodger H.D., Peric Z. (1996). Cell Culture Isolation of Piscine Neuropathy Nodavirus from Juvenile Sea Bass, Dicentrarchus Labrax. J. Gen. Virol..

[154] Zrnčić S., Padros F., Mladineo I., Fioravanti M.-L., Gustinelli A., Palenzuela O., Toffan A., Cuilli S., Fouz B., Breton A.L. (2022). Bottlenecks in Diagnostics of Mediterranean Fish Diseases.

[155] Baud M., Cabon J., Salomoni A., Toffan A., Panzarin V., Bigarré L. (2015). First Generic One Step Real-Time Taqman RT-PCR Targeting the RNA1 of Betanodaviruses. J. Virol. Methods.

[156] Sung C.-H., Lu J.-K. (2009). Reverse Transcription Loop-Mediated Isothermal Amplification for Rapid and Sensitive Detection of Nervous Necrosis Virus in Groupers. J. Virol. Methods.

[157] Xu H.-D., Feng J., Guo Z.-X., Ou Y.-J., Wang J.-Y. (2010). Detection of Red-Spotted Grouper Nervous Necrosis Virus by Loop-Mediated Isothermal Amplification. J. Virol. Methods.

[158] Hwang J., Suh S.-S., Park M., Oh M.-J., Kim J.-O., Lee S., Lee T.-K. (2016). Detection of Coat Protein Gene of Nervous Necrosis Virus Using Loop-Mediated Isothermal Amplification. Asian Pac. J. Trop. Med..

[159] Toffan A., Pascoli F., Pretto T., Panzarin V., Abbadi M., Buratin A., Quartesan R., Gijón D., Padrós F. (2017). Viral Nervous Necrosis in Gilthead Sea Bream (Sparus Aurata) Caused by Reassortant Betanodavirus RGNNV/SJNNV: An Emerging Threat for Mediterranean Aquaculture. Sci. Rep..

[160] Olveira J.G., Souto S., Dopazo C.P., Thiéry R., Barja J.L., Bandín I. (2009). Comparative Analysis of Both Genomic Segments of Betanodaviruses Isolated from Epizootic Outbreaks in Farmed Fish Species Provides Evidence for Genetic Reassortment. J. Gen. Virol..

[161] Panzarin V., Fusaro A., Monne I., Cappellozza E., Patarnello P., Bovo G., Capua I., Holmes E.C., Cattoli G. (2012). Molecular Epidemiology and Evolutionary Dynamics of Betanodavirus in Southern Europe. Infect. Genet. Evol..

[162] Biasini L., Berto P., Abbadi M., Buratin A., Toson M., Marsella A., Toffan A., Pascoli F. (2022). Pathogenicity of Different Betanodavirus RGNNV/SJNNV Reassortant Strains in European Sea Bass. Pathogens.

[163] Krishnan R., Kim J.-O., Qadiri S.S.N., Kim J.-O., Oh M.-J. (2020). Early Viral Uptake and Host-Associated Immune Response in the Tissues of Seven-Band Grouper Following a Bath Challenge with Nervous Necrosis Virus. Fish Shellfish. Immunol..

[164] Chen X., Qi J., He L., Luo H., Lin J., Qiu F., Wang Q., Zheng L. (2022). Isolation and Identification of a New Strain of Nervous Necrosis Virus from the Big-Belly Seahorse Hippocampus Abdominalis. Virol. J..

[165] Balakrishnan S., Singh I.S.B., Puthumana J. (2022). Status in Molluscan Cell Line Development in Last One Decade (2010–2020): Impediments and Way Forward. Cytotechnology.

[166] Lee L.E.J., Bufalino M.R., Christie A.E., Frischer M.E., Soin T., Tsui C.K.M., Hanner R.H., Smagghe G. (2011). Misidentification of OLGA-PH-J/92, Believed to Be the Only Crustacean Cell Line. In Vitro Cell. Dev. Biol. Anim..

[167] (2021). OIE CHAPTER 2.3.8 Infection with Salmon Alphavirus. Manual of Diagnostic Tests for Aquatic Animals.

[168] (2021). OIE CHAPTER 2.3.10 Infection with Viral Haemorrhagic Septicaemia Virus. Manual of Diagnostic Tests for Aquatic Animals.

